# AI-Powered Building Ecosystems: A Narrative Mapping Review on the Integration of Digital Twins and LLMs for Proactive Comfort, IEQ, and Energy Management

**DOI:** 10.3390/s25175265

**Published:** 2025-08-24

**Authors:** Bibars Amangeldy, Nurdaulet Tasmurzayev, Timur Imankulov, Zhanel Baigarayeva, Nurdaulet Izmailov, Tolebi Riza, Abdulaziz Abdukarimov, Miras Mukazhan, Bakdaulet Zhumagulov

**Affiliations:** 1 LLP «DigitAlem», Almaty 050042, Kazakhstan; bibars.amangeldy@ieee.org (B.A.); zhanel.baigarayeva@gmail.com (Z.B.); izmailov.nurdaulet@digitalem.kz (N.I.); riza.tolebi@digitalem.kz (T.R.); abdukarimov.abdulaziz@digitalem.kz (A.A.); mukazhan.miras@digitalem.kz (M.M.); zhumagulov.bakdaulet@digitalem.kz (B.Z.); 2 Faculty of Information Technology, Al-Farabi Kazakh National University, Almaty 050040, Kazakhstan

**Keywords:** smart buildings, artificial intelligence (AI), machine learning (ML), comfort, indoor environmental quality (IEQ), HVAC control, internet of things (IoT), sensors

## Abstract

Artificial intelligence (AI) is now the computational core of smart building automation, acting across the entire cyber–physical stack. This review surveys peer-reviewed work on the integration of AI with indoor environmental quality (IEQ) and energy performance, distinguishing itself by presenting a holistic synthesis of the complete technological evolution from IoT sensors to generative AI. We uniquely frame this progression within a human-centric architecture that integrates digital twins of both the building (DT-B) and its occupants (DT-H), providing a forward-looking perspective on occupant comfort and energy management. We find that deep reinforcement learning (DRL) agents, often developed within physics-calibrated digital twins, reduce annual HVAC demand by 10–35% while maintaining an operative temperature within ±0.5 °C and CO_2_ below 800 ppm. These comfort and IAQ targets are consistent with ASHRAE Standard 55 (thermal environmental conditions) and ASHRAE Standard 62.1 (ventilation for acceptable indoor air quality); keeping the operative temperature within ±0.5 °C of the setpoint and indoor CO_2_ near or below ~800 ppm reflects commonly adopted control tolerances and per-person outdoor air supply objectives. Regarding energy impacts, simulation studies commonly report higher double-digit reductions, whereas real building deployments typically achieve single- to low-double-digit savings; we therefore report simulation and field results separately. Supervised learners, including gradient boosting and various neural networks, achieve 87–97% accuracy for short-term load, comfort, and fault forecasting. Furthermore, unsupervised models successfully mine large-scale telemetry for anomalies and occupancy patterns, enabling adaptive ventilation that can cut sick building complaints by 40%. Despite these gains, deployment is hindered by fragmented datasets, interoperability issues between legacy BAS and modern IoT devices, and the computer energy and privacy–security costs of large models. The key research priorities include (1) open, high-fidelity IEQ benchmarks; (2) energy-aware, on-device learning architectures; (3) privacy-preserving federated frameworks; (4) hybrid, physics-informed models to win operator trust. Addressing these challenges is pivotal for scaling AI from isolated pilots to trustworthy, human-centric building ecosystems.

## 1. Introduction

Buildings remain the largest end-use energy consumer, responsible for roughly one-third of global final demand and about one-quarter of anthropogenic CO_2_ emissions [1,2,3]. Because people spend up to 90% of their time indoors, IEQ directly affects physiology, cognition, and economic output. Indoor environmental quality (IEQ)—the combined thermal, acoustic, visual, and air quality conditions—has measurable effects: CO_2_ increases from 600 to 1 000 ppm slow decision-making by ~15%, and ±2 °C thermal deviations correlate with higher complaints and absenteeism [4,5]. Balancing energy demand with IEQ is therefore a multi-objective optimization challenge.

The engineering toolkit for tackling that challenge has progressed through several eras. The 1970s introduced centralized building automation systems (BAS) that applied closed-loop control and delivered tangible energy savings [4]. Scientometric analyses show that the interest in smart building research has accelerated markedly in the past decade [6], and systematic reviews underline the persistent integration challenges and opportunities that accompany this growth [7]. From the early 2020s, low-power wireless protocols such as Zigbee, Thread, and LoRaWAN reshaped BAS into IoT-centric platforms capable of interconnecting thousands of sensing and actuation points [8].

A fourth, emerging era layers artificial intelligence (AI) onto this IoT fabric. Reviews trace the evolution of AI techniques aimed at boosting building energy efficiency [9] and show how machine learning models coupled with IoT data streams advance that goal [10], while field implementations demonstrate intelligent control that reduces energy use without compromising comfort [11]. When multimodal occupancy sensing—passive infrared, ultrasound, Wi-Fi analytics, and computer vision—is added to the mix [12], sequence models forecast presence patterns with mean absolute errors below 10% [13], providing a foundation for model-predictive and reinforcement learning controllers that modulate HVAC equipment proactively.

These capabilities typically reside in a three-layer architecture. The perception layer hosts modular nodes for temperature, humidity, illuminance, CO_2_, acoustics, and motion; the cognitive layer embeds gradient boosting, convolutional, and graph neural networks, refining digital twin simulations with sub-room granularity; and the control layer executes multi-objective optimization constrained by predicted occupancy and weather. Hybrid physical–statistical baselines reduce CV-RMSE by up to 8% relative to classical regression [14], and urban-scale extensions that account for morphology, albedo, and micro-climate improve heat flow assessment across districts [15]. Parallel work on occupant-centric metrics links control decisions to subjective comfort and disease prevention [16], and the publication of large, open datasets is accelerating the replication and benchmarking of these approaches [17].

To ground the discussion, narrative literature mapping was performed across the Web of Science, Scopus, and IEEE Xplore databases. The search used combinations of keywords including “smart building”, “indoor environmental quality”, “energy efficiency”, “machine learning”, “IoT”, “comfort”, “health”, and “HVAC control”. We retained peer-reviewed articles published from 2015 to 2025 that explicitly link building energy consumption to IEQ, comfort, or health, while excluding gray literature, duplicates, and studies with non-transparent methodologies. The resulting corpus of 180 publications provides the evidentiary foundation for the analysis that follows. The context of climate change further highlights the urgency of implementing such integrated solutions [18].

This review offers an integrative synthesis across building sensing, digital twins, AI-based HVAC control, and emerging LLMs for building management systems (BMS), consolidating segmented surveys rather than claiming algorithmic novelty. Specifically, we frame the literature along an end-to-end sequence—sensors → digital twin → AI control → LLMs in BMS. By mapping interfaces, data handoffs, and evaluation metrics across these layers, this review bridges strands that are often treated in isolation and surfaces system-level gaps (occupancy inference-to-control feedback, twin calibration-to-policy transfer, and LLM grounding in BMS ontologies). In contrast to specialized studies, our paper traces the full evolution—from sensors to generative AI—within a single, human-centric architecture. We introduce the integrated concept of digital twins of the building and the human (DT-B + DT-H) and analyze how advanced AI models, including LLMs, enable the shift from reactive control to a proactive and hyper-personalized environment aimed at maintaining health and comfort.

The next parts present recent statistics on energy use alongside findings that link IEQ to health and productivity. The section titled “Ecosystem of Sensors, Data, and Predictive Intelligence” then examines the shift from legacy building automation systems (BAS) to IoT-enabled buildings and details the architecture and algorithms that deliver proactive comfort and health management. A dedicated methodology section elaborates on the narrative mapping process, followed by a discussion of outstanding challenges and practical recommendations. The conclusion synthesizes the findings and outlines avenues for future work. The remainder of the paper follows this pipeline to maintain conceptual continuity and highlight cross-layer dependencies.

## 2. Methodology

**Figure 1:**
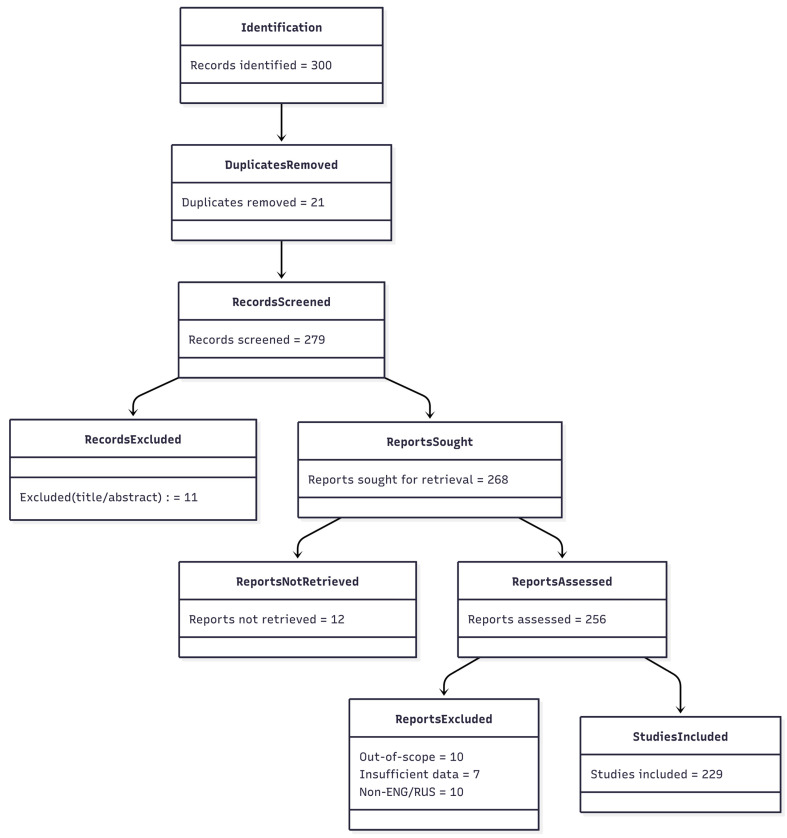
PRISMA-ScR flow of study selection.

To frame the evidence synthesis and ensure transparent, reproducible reporting, the study selection process followed the PRISMA-ScR guideline and is summarized in Figure 1. Of the 300 records initially identified, 21 duplicates were removed, leaving 279 publications for title/abstract screening; at this step, 11 records were excluded, and full texts were sought for 268 articles. However, 12 full texts could not be retrieved, resulting in 256 reports assessed for eligibility. Following full text assessment, a further 27 publications were excluded for different reasons (out of scope—10; insufficient data—7; non-ENG/RUS—10), and 229 studies were included in the analysis. Screening was performed by two independent reviewers, with disagreements resolved by a third expert; the search window was 2009–2025; sources: Scopus, Web of Science, Nature, IEEE Xplore, ACM Digital Library, PubMed, and Google Scholar. Thus, the sequence of stages from “Identification” to “Studies included” and the counts at each step are fully consistent, as shown in Figure 1.

The distribution of included sources aligns with the review structure. Section 1 (Introduction) comprises 18 papers covering sectoral statistics, the direct link between IEQ and health/well-being/productivity, the significant energy footprint of buildings, and the transition from traditional buildings to BAS and then to IoT-enabled smart buildings; it also includes overview pieces on AI for smart buildings (architecture “building + comfort + health + AI”) and methodological notes (databases, keywords, and publication window). Section 3 (Ecosystem of Sensors, Data, and Predictive Intelligence) aggregates 75 studies on how the architectural layers work together (IoT layer → communication → processing and control → actuation), on multimodal sensing (incl. smartwatches, fitness trackers, and smartphones), and on the personal comfort and health digital twin concept. Section 4 (Artificial Intelligence for Prediction, Control, and Interaction) includes 107 studies spanning foundational ML (supervised/unsupervised), advanced AI for adaptive control, reinforcement learning for HVAC control, generative AI and LLMs for comfort/health management, and personalization via fine-tuning. Section 5 (Discussion) synthesizes 29 publications focusing on data quality and scarcity, the robustness and generalization of fine-tuned models, cybersecurity risks, the paradox of control and loss of agency, privacy, unintended health consequences of over-optimization, ethical and societal implications, and future trajectories/solutions.

Table 1 consolidates the essential design and outcome information across the reviewed studies, covering building and climatic context, sensing and control stacks, analytical and optimization methods, data provenance, baseline configurations, metric definitions and validation procedures, the experimental scenario (simulation, field, or testbed), and a concise appraisal of costs and benefits. Where studies report comparable quantities, ranges or absolute changes (e.g., energy use, reliability, latency) are included. Missing details are marked “not reported.” Bracketed numerals map to the reference list.

## 3. Ecosystem of Sensors, Data, and Predictive Intelligence

Smart buildings have evolved from simple collections of stand-alone, hard-wired controls into richly instrumented, data-driven ecosystems that sense, think, and act in real time [21,26]. At the foundation of this ecosystem sits a dense fabric of environmental sensors—temperature, humidity, CO_2_, and illuminance—and occupancy detectors ranging from passive infrared (PIR) and image-based sensors to Bluetooth beacons [27]. These devices stream time-stamped telemetry into an Internet-of-Things (IoT) network that blends legacy building automation protocols with low-power wireless standards like Zigbee and LoRaWAN, guaranteeing a secure communication between devices and controllers [26,28].

The first computational stop for these data is the edge layer, where embedded gateways perform noise filtering and low-latency inference [20]. Edge-resident machine learning models can predict imminent threshold violations—say, a rapid rise of CO_2_ in a crowded meeting room—and trigger timely corrective actions while circumventing round-trip delays to the cloud [20].

Cleaned and summarized datasets flow onward to cloud platforms that offer virtually unlimited storage and processing power [29,30]. Here, historical telemetry is fused with external variables like weather forecasts and dynamic electricity tariffs [31]. The same cloud tier hosts holistic digital twins: data-driven replicas that couple Building Information Model (BIM) geometry with live sensor feeds [32,33]. These twins enable what-if experiments, fault diagnostics, and model-predictive control routines that optimize comfort, air quality, and energy consumption simultaneously [32,34].

At the application layer, operators and occupants interact with the system through dashboards, mobile apps [35], and, increasingly, conversational agents underpinned by large language models (LLMs) [36,37]. A facilities manager can visualize thermal maps and equipment health indices, while an employee can nudge personal set-points or receive notifications.

The closed-loop interaction unfolds as follows. Sensors perceive the indoor environment and occupant presence [22]; edge analytics detect patterns [23]; predictive models in the cloud forecast future states [26]; optimization engines compute control trajectories that balance comfort, health, and energy costs [30]; and actuators in HVAC and lighting systems execute the commands [24]. Throughout, cybersecurity and privacy measures—device authentication, encrypted channels, and privacy-preserving federated learning—safeguard the integrity of both data and people [23,33].

Several concrete scenarios illustrate the value of this architecture. Demand-controlled ventilation systems use CO_2_ predictions to increase airflow minutes before concentrations reach cognitive performance limits [23]. Behavior-aware HVAC scheduling mines occupancy data to pre-condition spaces only when needed, trimming peak loads [34,35]. Multi-objective reinforcement learning agents, trained inside digital twins, can orchestrate environmental controls to cut electricity bills significantly [35,36]. In safety-critical events such as fires, the same sensor and control backbone can automatically shut down air handling units and unlock egress routes [28].

By intertwining pervasive sensing, reliable data pipelines, and predictive intelligence across edge and cloud domains [25], modern smart building ecosystems transcend reactive automation and deliver proactive, human-centric environments [9]. Occupants benefit from healthier air and greater comfort, while owners realize significant gains in energy efficiency, maintenance planning, and asset resilience [9,10].

**Figure 2:**
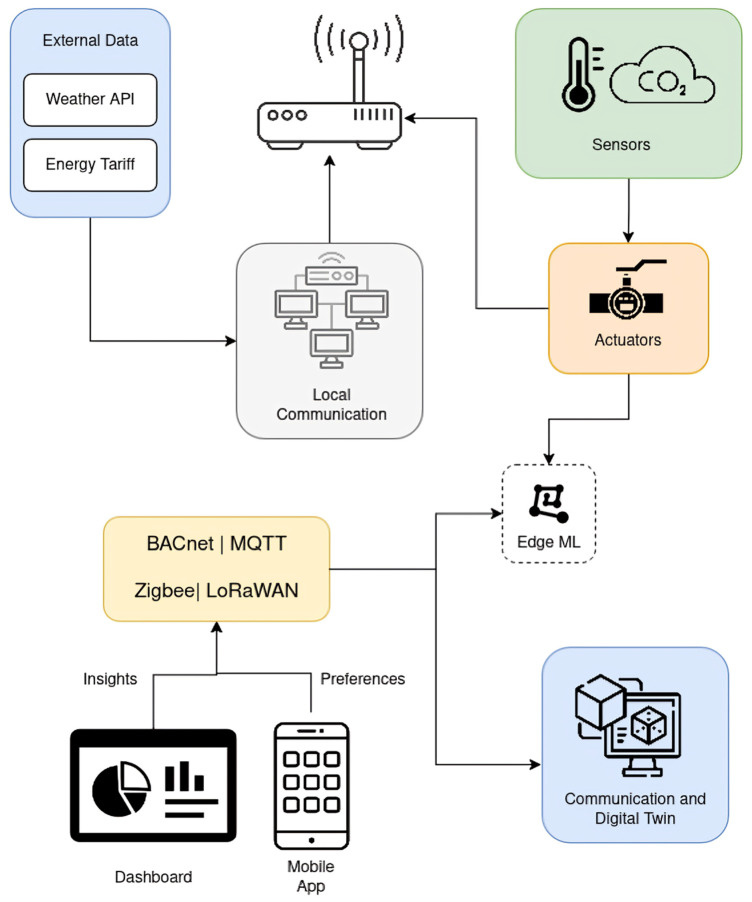
Ecosystem of sensors, data, and predictive intelligence in a smart building.

The realization of proactive, human-centric control is underpinned by a multi-layered cyber–physical architecture that orchestrates the cyclical flow of data from the physical environment to cloud-based intelligence and back to physical actuators (illustrated in Figure 2). This integrated framework is designed to sense, reason, and act in real-time, transforming raw telemetry into intelligent control actions that continuously optimize for occupant comfort, health, and energy efficiency.

At its foundation lies the perception and actuation layer, which constitutes the direct interface with the building and its occupants. A dense fabric of environmental sensors (for temperature, humidity, and CO_2_) and multimodal occupancy detectors—ranging from passive infrared (PIR) to advanced millimeter wave (mmWave) radar—generates a continuous stream of high-granularity telemetry [21,22,31]. Conversely, actuators within HVAC systems, lighting, and automated shading execute the control commands that physically alter the indoor environment.

This raw data is transmitted upwards through the communication and IoT layer. This layer leverages a hybrid of legacy wired protocols like BACnet and modern wireless standards such as Zigbee and LoRaWAN to ensure reliable and secure connectivity between the physical devices and the computational layers [20,22,33]. The first computational stop for this data stream is the edge computing and local layer. Here, local gateways and embedded machine learning (ML) modules perform critical low-latency functions, including data filtering, aggregation, and immediate inference for time-sensitive responses. This stage is crucial for immediate actions and for reducing the data load on the cloud infrastructure [24,25].

Cleaned and pre-processed data, often fused with external data from sources like weather APIs and dynamic energy tariffs [26], proceeds to the cloud analytics and digital layer. This central intelligence hub hosts scalable data lakes for long-term storage and automated machine learning (AutoML) platforms for developing predictive models [25]. The core of this layer is the digital twin—a dynamic, physics-informed virtual replica of the building continuously synchronized with real-world sensor feeds. This twin serves as an invaluable sandbox for simulating complex scenarios, performing fault diagnostics, and training reinforcement learning agents for multi-objective optimization [27,29].

Finally, the applications and UI layer manages the interaction with human stakeholders. Through intuitive interfaces like dashboards, mobile applications, and conversational agents, facility managers and occupants receive actionable insights derived from the cloud analytics [19,32]. Crucially, this layer also allows users to provide direct feedback and explicit preferences, creating a human-in-the-loop control system that is vital for personalization and closing the reinforcement cycle [19].

The entire architecture operates as a continuous, closed loop. Data flows upward from sensors to the cloud, where it is transformed into intelligent decisions. These decisions propagate downwards as control signals to actuators. The system’s adaptive capability is perpetually reinforced by feedback mechanisms, including model updates sent from the cloud back to the edge layer, ensuring it learns and evolves over time. This synergistic integration of sensing, connectivity, and predictive intelligence is what creates a truly proactive, resilient, and human-centric building ecosystem.

### 3.1. Environmental Sensing Technologies

The proactive management of comfort and health in smart buildings relies on a multi-layered technological architecture. This framework provides the essential structure for sensing the environment, communicating data, processing information, and enacting physical changes. This section deconstructs this architecture by outlining its operational workflow, presenting a visual model, and analyzing its role in current research and future development.

The effectiveness of smart building systems hinges on the comprehensive and accurate data gathered by the sensing layer, which comprises a variety of IoT environmental sensors designed to capture real-time information about the indoor environment. Studies on indoor air quality (IAQ) in sensitive environments, such as daycare centers, emphasize the important role of IoT sensors in monitoring parameters like temperature, humidity, and CO_2_ levels to ensure safe and healthy conditions for occupants [37]. Beyond environmental parameters, the sensing layer also extends to energy consumption monitoring, with industrial IoT (IIoT)-based submetering solutions deploying IoT-enabled submeters to provide real-time energy consumption data from critical equipment, enabling optimized energy management and waste reduction in manufacturing facilities [38]. Similarly, for building equipment energy saving optimization, online monitoring systems leverage IoT sensors to collect data that, when integrated with Building Information Modeling (BIM), allows for the intelligent control of systems like air conditioners [39]. The optimal design of communication topology for wireless sensor networks (WSNs) is also crucial for efficient data collection, considering factors like network energy consumption and stability to implement fully distributed optimal control approaches in IoT-enabled smart buildings [40].

The communication layer is responsible for the reliable and efficient transmission of data from sensors to processing units and control signals to actuators, employing various wireless and wired technologies. The communication layer is responsible for the reliable and efficient transmission of data from sensors to processing units, as well as control signals to actuators, using various wired and wireless technologies. LPWAN (Low-Power Wide-Area Network) technologies are gaining increasing popularity due to their ability to transmit data over long distances with low energy consumption, making them ideal for many IoT applications in smart buildings [41]. Furthermore, the integration of advanced networking paradigms like Named Data Networking (NDN) with edge computing can significantly enhance IoT performance in wireless and mobile networks by optimizing data retrieval and caching, thereby reducing latency and improving reliability [42]. This focus on efficient communication protocols is vital for supporting the real-time demands of smart building operations.

The processing layer is where raw sensor data is transformed into actionable insights through advanced computational techniques, often involving a combination of edge computing and cloud-based analytics. Edge computing allows for localized data processing, reducing latency and bandwidth requirements, which is particularly beneficial for real-time IoT applications [42]. For more complex analyses and long-term data storage, cloud integration remains essential. The development of decentralized machine learning frameworks for IoT is also enhancing security, privacy, and efficiency in these cloud-integrated environments [43]. The architectural flexibility of microservices-based IoT platforms is a key enabler in this layer, allowing for scalable, interoperable, and dynamic ecosystems that can efficiently handle the distributed nature of IoT devices [44]. Moreover, machine learning techniques are increasingly employed within this layer for critical tasks such as attack detection in IoT networks, bolstering the cybersecurity posture of smart building systems [45]. The final layer, actuation and control, translates the intelligent decisions made by the processing layer into physical changes within the building environment. This involves sending commands to various devices, such as HVAC systems, lighting controls, and air purifiers, to adjust conditions according to desired comfort, health, and energy efficiency parameters. The continuous feedback loop from the sensing layer allows the system to monitor the effects of these actions and refine its control strategies, leading to adaptive and optimized building performance.

The architectural structure of smart buildings is not isolated but operates within a broader Internet of Things (IoT) ecosystem. This wider context includes initiatives aimed at creating open IoT innovation ecosystems for smart cities, which focus on ensuring interoperability through open communication and data standards [46,47]. These principles also extend to the industrial sector, where IoT smart factory ecosystems are being developed based on Software-Defined Networking (SDN) to enhance communication and increase efficiency in industrial processes [48].

A key aspect across all levels of this architecture is security. As the number of IoT devices grows, so does the number of potential vulnerabilities, prompting ongoing research focused on developing countermeasures and building robust security systems [49]. Additionally, the sustainability of widespread IoT deployment is becoming an increasingly important issue, driving interest in energy supply solutions. Thermoelectric Generators (TEGs) are one promising option for powering autonomous sensors, converting thermal gradients into electrical energy and thereby supporting their self-sufficiency while reducing the environmental footprint of smart building components [50]. The ongoing evolution of this multi-layered architecture, coupled with advancements in AI, communication technologies, and sustainable power solutions, promises to deliver increasingly intelligent, responsive, and resilient smart buildings that proactively manage occupant comfort and health.

Table 2 provides an overview of the sensor and IoT technologies used in smart buildings, including measured parameters, areas of application, and advantages. The first technology is indoor air quality sensors (IAQ sensors), which measure temperature, humidity, and CO_2_ levels. They are used for monitoring air quality in sensitive areas such as medical facilities or laboratories and provide data on air conditions to maintain safe environments. The second technology is industrial IoT, designed for measuring and optimizing energy consumption in industrial buildings. It enables real-time monitoring, energy resource management, and a reduction in energy losses. The third technology is IoT sensors integrated with Building Information Modeling (BIM). These sensors collect data aimed at improving equipment energy efficiency and are used for the online monitoring and automatic control of building systems. The fourth technology is wireless sensor networks (WSNs), which collect data for implementing distributed control. They are used for data acquisition in smart buildings and support the development of communication topologies that consider energy consumption and system stability. All listed technologies are supported by references to scientific sources.

### 3.2. Personal and Occupancy Sensing Technologies

The cornerstone of any system designed to proactively manage comfort and health is the ability to collect accurate, non-stop data about the people in a building. Choosing the right sensing technology, however, is a major challenge, forcing a trade-off between how detailed the data is, how much it costs to implement, and, most critically, how much it invades occupant privacy. This section offers a deep dive into the key sensing technologies, assessing how they fit into the main goal of creating personalized, healthy, and comfortable spaces.

The first layer of data collection comes from ambient (or occupational) sensors placed within a space. The most basic of these, passive infrared (PIR) detectors, are effective for simply detecting presence, but their binary “present/not present” logic cannot assess the number of people or their metabolic activity, which limits their use in advanced HVAC systems [51]. Computer vision (CV)-based systems offer the highest level of detail: they can accurately count people, identify their postures, and determine activity levels, which are all direct inputs for thermal comfort (PMV) calculation models. However, their use is associated with severe privacy violation concerns, making them unacceptable for most residential and office environments [52].

To solve this dilemma, privacy-preserving technologies have been developed. Wi-Fi Channel State Information (CSI) analysis uses existing infrastructure to estimate occupancy with reasonable accuracy [53], but its performance can be unstable due to changes in the environment [54]. A more robust alternative is millimeter wave (mmWave) radar. These devices do not create images but can determine the number and location of people with high precision, as well as detect micro-motions like respiration rate, which is directly relevant for assessing air quality and health status [55,56].

However, to transition from group-level to personalized management, wearable devices are essential, as they provide physiological data that is inaccessible to ambient sensors. These devices track key parameters such as Heart Rate (HR) and Heart Rate Variability (HRV), which are reliable proxies for metabolic rate (heat production) and stress levels, enabling control systems to deliver personalized cooling or adapt lighting to reduce stress [57]. In addition, skin temperature offers a direct indicator of a person’s thermal balance, allowing the system to react more quickly to individual discomfort. Activity levels, captured via an accelerometer, are also a critical parameter for dynamically calculating metabolic rate in thermal comfort models like PMV [58].

A comparison of wearable devices reveals that while budget-friendly trackers (Xiaomi Mi Band) are suitable for general activity assessment, more expensive devices (Oura Ring, Apple Watch) provide more accurate temperature and HRV data, which is preferable for sophisticated health and stress models [59].

As it turns out, no single technology is a perfect solution by itself. Therefore, the most sensible approach is sensor fusion—combining data from multiple sources. For example, a system might use an mmWave radar to accurately count people in a zone, while data from their wearables determines the average metabolic load for that zone. This allows the HVAC system to be both energy-efficient and highly personalized to individual needs. Table 3 summarizes this comparative analysis, contrasting the primary technologies based on their key characteristics and relevance for comfort and health management systems. The data acquired from this multi-layered sensor network serves as the foundation for the digital twin models discussed in the next section.

In this review, thermal comfort is operationalized using the PMV/PPD and/or adaptive model, with compliance expressed as the proportion of occupied hours within the ASHRAE 55 acceptable zone (approximately PMV −0.5…+0.5, i.e., ≤10% PPD) [60,61]. Indoor air quality (IAQ) adequacy is assessed according to the Ventilation Rate Procedure (VRP) specified in ASHRAE 62.1; no fixed indoor CO_2_ limit is imposed, with CO_2_ instead applied as a proxy indicator for ventilation performance [62,63,64]. In steady-state conditions, indoor CO_2_ levels approximately 600–800 ppm above the outdoor baseline are used as a practical indicator of insufficient ventilation, guiding demand-controlled ventilation (DCV) settings in conjunction with space-specific CO_2_ metrics [62,63]. These anchors enable the direct linkage of sensor data streams to measurable comfort and IAQ targets, which are then applied in the subsequent control and evaluation stages [60,62,65].

In Table 4 we demonstrated the detailed mapping of key sensor modalities to their primary measurable metrics, associated standard anchors, representative multimodal fusion strategies, and trade-offs across cost, energy consumption, privacy, and bias dimensions. The “Primary metric(s) mapped” column specifies the measurable outputs linked to comfort (e.g., operative temperature, and PMV/PPD), IAQ (e.g., CO_2_ as a ventilation adequacy proxy), or ergonomics (e.g., acoustic noise levels). “Standard anchor” identifies the normative framework or procedural logic—such as ASHRAE Standard 55 for thermal comfort or ASHRAE Standard 62.1 [66] VRP for ventilation—used to interpret each metric in a building performance context. “Typical fusion” lists common multimodal combinations (e.g., CO_2_ + PIR/mmWave for robust demand-controlled ventilation, Temp + RH + air speed for PMV computation, and mmWave + wearables for metabolic load estimation) that enhance the accuracy and resilience to single sensor limitations. The “Key trade-offs” column summarizes practical considerations that influence deployment decisions, including initial and operational costs, energy overhead, data privacy implications, and inherent biases or sensing limitations. This synthesis supports the review’s objective to link sensing technologies explicitly to measurable targets, applicable standards, and integrative control strategies for occupant-centric and energy-efficient building management.

### 3.3. Digital Twin Technology

The data gathered from this sensor network serves as the foundation for the next leap in building management: digital twin (DT) technology. A DT is a living virtual replica of a physical asset, synchronized with it in real time. In this context, as outlined in foundational reviews [68,69], the concept is split into two interconnected entities: the digital twin of the building (DT-B) and the digital twin of the human (DT-H).

A DT-B is a dynamic, physics-based model of the building. For over a decade, the consensus has been that “gray-box” models, which blend physical principles with data-driven AI, are optimal [70]. The historical challenge, however, has been making them fast enough for live control. Recent research has tackled this problem from different angles, addressing key bottlenecks that previously hindered real-time operation. For instance, to solve the problem of calibration speed, a 2024 framework called GenPhysiCal can perform a full calibration cycle in just 0.04 s [71]. In parallel, to overcome the challenge of simulation complexity, a 2025 study uses an AI-based “surrogate model” to predict complex airflow in milliseconds instead of minutes, maintaining a high accuracy [72]. Together, these advances make real-time DT-B operation feasible.

A fast, accurate DT-B provides a ‘virtual sandbox’ for learning control policies. Comparing the evolution of this approach from classical MPC [70] to modern AI shows rapid progress. A foundational 2019 review [73] and subsequent application papers [74] showed that deep reinforcement learning (DRL) could achieve significant energy savings of 15–40%. More recently, a 2024 study using Bayesian optimization demonstrated a more holistic benefit: it improved occupant comfort by 38% while simultaneously reducing energy consumption [75]. This highlights a critical shift in research goals, moving from a pure energy focus to a dual-objective approach that balances efficiency with human well-being.

Parallel to the DT-B, the concept of the human digital twin (DT-H) is evolving. While the DT-H is already a major topic in personalized medicine for testing treatments in silico, as shown in recent 2024 reviews [76,77], its role in smart buildings is to model an individual’s comfort. This is achieved by feeding biometric signals from wearables into sophisticated thermoregulation models. For example, the Fiala model can be parameterized in real time with wearable data to predict personal thermal sensation, a method demonstrated by Al-Khafaji et al. [78].

**Figure 3:**
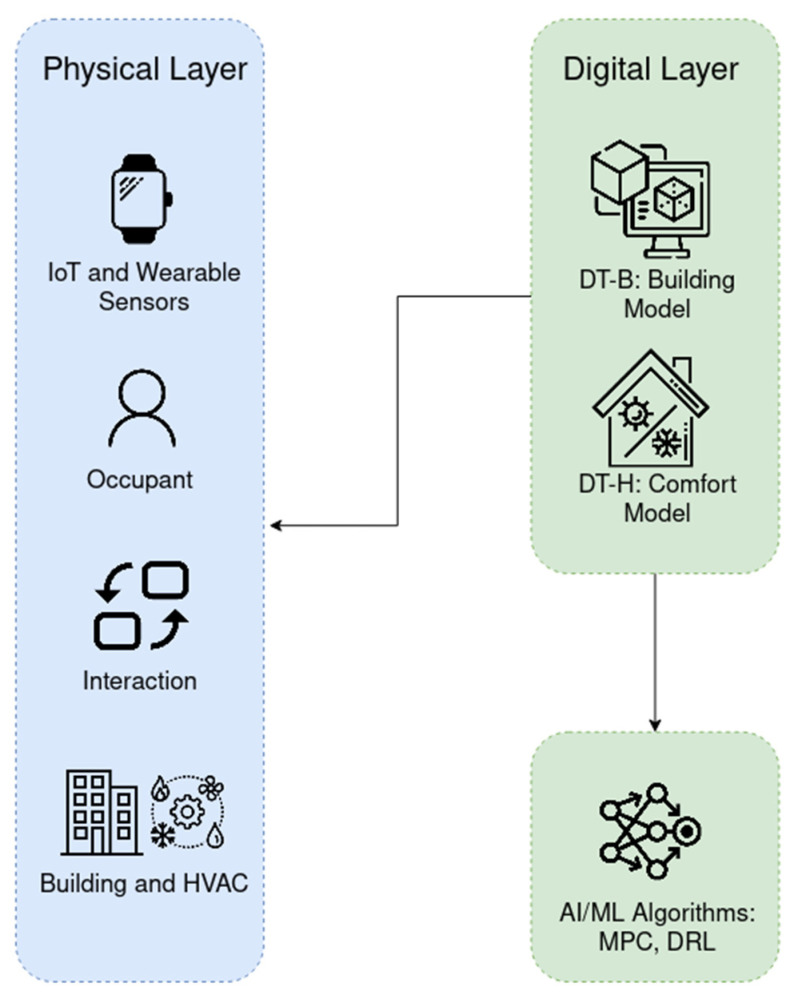
Conceptual architecture of a hybrid digital twin (DT-B + DT-H) for proactive occupant comfort.

The culmination of this technology is the hybrid “building–human” twin, which links the DT-B and the DT-H, as shown in Figure 3. This architecture materializes the “human-in-the-loop” control concept reviewed by Papantoniou et al. [79], where a person’s physiological state (from the DT-H) directly informs the building’s control systems (the DT-B). This is no longer just a theoretical concept; a 2024 pilot study on a Korean smart campus provided hard evidence, demonstrating that this hybrid system increased the time occupants spent in their individual comfort zone from 62% to an impressive 85% [80].

Despite this significant potential, widespread implementation faces hurdles. As a major 2024 systematic review by El-Amroussi et al. confirms, challenges like the interoperability between different models and the standardization of data exchange remain critical barriers [81]. Nevertheless, the digital twin architecture provides the necessary infrastructure to treat thermal comfort, air quality, and health not as separate challenges, but as a single, dynamically optimized objective function.

Interoperability and semantic standards such as brick ontology and IFC (ISO 16739) [82] have emerged as critical enablers for seamless digital twin integration in the built environment. A recent analysis of IFC-based workflows for embedding Environmental Product Declaration (EPD) data illustrates that, despite its comprehensive schema, semantic alignment challenges persist when integrating Life Cycle Assessment (LCA) information into Building Information Modeling (BIM) and digital twins [83]. Addressing these gaps requires not only standardized data models but also structured ontologies capable of supporting cross-domain integration. In this context, multi-domain ontologies anchored in IFC have been proposed as a basis for incremental digital twin conceptualizations [84]. Beyond structural information, semantic web technologies have been applied to domains such as indoor environmental quality, where the IFC ontology structure is complemented by frameworks like the Smart Applications REFerence ontology (SAREF) to enhance semantic interoperability and data reuse in building performance monitoring [85]. At the asset end-of-life stage, reviews of BIM-based digital deconstruction approaches reveal that ontologies accepting IFC inputs can streamline demolition planning, material recovery, and reuse processes, supporting sustainability objectives within digital twin environments [86]. This interoperability imperative also extends to infrastructure, where strategies for reinforced concrete bridge management in compliance with Italian regulations emphasize open formats such as IFC to maintain compatibility between inspection data, 3D modeling, and maintenance systems [87].

Cultural heritage contexts further highlight the role of semantic standards. Heritage BIM (HBIM) approaches for twentieth-century concrete structures leverage IFC as a foundation for integrating geometric documentation, historical metadata, and sensor data into coherent digital twins [88]. In a similar way, HBIM workflows for built heritage utilize IFC to support interoperable virtual and augmented reality applications, fostering wider access and collaboration among stakeholders [89]. Interoperability is equally relevant in operational management, where integrating BIM, Internet of Things (IoT), and facility management systems through semantic construction digital twins—structured around IFC—addresses the challenges of linking real-time sensor data with as-built models [90]. Systematic reviews of BIM-based structural health monitoring confirm that ISO 16739-compliant IFC schemas facilitate sensor data integration, enabling the continuous monitoring of assets such as historical churches and masonry bridges [91]. The role of IFC in enhancing stakeholder collaboration is underscored in studies on BIM-driven sustainable heritage tourism, where embedding semantic information into IFC models supports richer, more accessible cultural heritage experiences [92]. These works demonstrate that adherence to semantic standards such as IFC—complemented where appropriate by domain-specific ontologies—remains central to achieving the full interoperability potential of digital twins across building, infrastructure, and heritage domains.

## 4. Artificial Intelligence for Prediction, Control, and Interaction

### 4.1. Foundational Machine Learning

Machine learning has emerged as the cornerstone technology for intelligent building management, delivering unprecedented energy savings of 15–50% while maintaining optimal occupant comfort [92,93]. The transformation from traditional rule-based building control to adaptive machine learning systems represents a fundamental shift in how buildings respond to dynamic environmental conditions, occupancy patterns, and energy demands [94]. This technological evolution addresses the critical challenge that buildings consume approximately 30–34% of global energy consumption [95,96], making efficient building operation essential for sustainability goals.

The foundational machine learning approaches in smart buildings encompass both supervised and unsupervised learning paradigms, each serving distinct but complementary roles in building intelligence. Supervised learning algorithms excel at prediction tasks, achieving 92–97% accuracy in energy forecasting and occupancy detection, while unsupervised learning methods reveal hidden patterns in building data, enabling fault detection and operational optimization without requiring labeled training data [97,98]. Recent research demonstrates that machine learning implementations in commercial buildings consistently outperform traditional control systems, with ensemble methods achieving the highest accuracy rates of 95–98% for energy prediction tasks [99].

#### 4.1.1. Supervised Learning Technologies for Smart Buildings

Supervised learning forms the predictive foundation of intelligent building systems, leveraging historical data to forecast energy consumption, occupancy patterns, and equipment performance. Support Vector Machines (SVMs) and Support Vector Regression (SVR) have demonstrated exceptional performance in building energy prediction, achieving prediction accuracies with CV less than 3% and percentage errors within 4% for long-term electricity consumption forecasting [100]. Li et al. established that SVM consistently outperforms Artificial Neural Networks (ANNs) for building energy prediction, with a Root Mean Square Error (RMSE) of 7.35 compared with ANN’s 5.71 for office building applications [101]. Decision trees with advanced algorithms like CART and C4.5 demonstrate exceptional interpretability while maintaining a high accuracy for building design optimization. Ref. [102] achieved 93.5% classification accuracy for zero energy building design using 15 passive design parameters, providing transparent decision-making processes crucial for regulatory compliance.

Building on SVM’s strong foundation, ANNs represent one of the most versatile supervised learning approaches for smart buildings, consistently achieving the highest accuracy for heating and cooling load prediction across diverse building types [36]. Recent implementations demonstrate that neural networks deliver 67% accuracy for thermal comfort prediction and superior performance compared with engineering calculation methods for building energy consumption [103,104]. Deep learning architectures, particularly CNN-LSTM hybrids, represent the cutting edge of neural network applications in buildings. Ref. [105] achieved a near-perfect prediction performance (R^2^ > 0.99) by combining CNN spatial feature extraction with LSTM temporal modeling for residential energy forecasting.

Complementing the high accuracy of individual neural networks, Random Forest and ensemble methods have emerged as the optimal balance between accuracy and interpretability for building applications. Research demonstrates Random Forest achieving 96.7% accuracy for thermal comfort prediction using PMV values, while maintaining R^2^ = 0.92 with RMSE = 360.17 for European energy consumption prediction [106]. The algorithm’s ability to handle multiple input variables and provide feature importance rankings makes it particularly valuable for building energy analysis, where operators need to understand which factors most influence building performance. Advanced ensemble methods beyond traditional Random Forest show superior computational efficiency. Ref. [107] demonstrated that LightGBM optimized with Satin Bowerbird Optimizer achieved R^2^ = 0.9148, significantly outperforming Random Forest (R^2^ = 0.8902) for building energy prediction tasks.

Further advancing ensemble methodologies beyond Random Forest, XGBoost (Extreme Gradient Boosting) has established itself as the premier algorithm for structured building data, consistently achieving 96.4% accuracy for thermal comfort modeling and demonstrating superior performance across multiple building applications [108]. The algorithm’s built-in regularization and ability to handle missing data make it particularly suitable for real-world building datasets, where sensor malfunctions and data gaps are common challenges. Probabilistic approaches like Naive Bayes combined with preprocessing techniques offer computational efficiency for categorical building tasks. Ref. [109] showed that integrating K-means clustering with Naive Bayes classification significantly improves HVAC energy consumption prediction accuracy compared with standalone implementations.

These supervised learning approaches demonstrate a clear evolution from individual algorithms like SVM and ANN to sophisticated ensemble methods like Random Forest and XGBoost, each building upon the strengths of previous approaches while addressing their limitations. To further illustrate these advancements, Table 5 offers a comparative summary of each algorithm’s primary applications, performance metrics, and unique benefits within smart building contexts.

#### 4.1.2. Unsupervised Learning Technologies for Smart Buildings

Unsupervised learning technologies enable buildings to discover hidden patterns and anomalies without requiring labeled training data, making them essential for fault detection, energy optimization, and operational pattern recognition. K-means clustering has demonstrated exceptional effectiveness for energy consumption pattern analysis, achieving 89.3% accuracy in electricity use pattern categorization for university buildings while successfully identifying distinct operational patterns including base consumption, human activity consumption, and HVAC consumption [110]. The algorithm’s ability to segment building energy signatures enables facility managers to identify inefficiencies and optimize operational schedules based on actual usage patterns. Density-based clustering methods like DBSCAN demonstrate superior performance for non-spherical energy consumption patterns and anomaly detection. Recent implementations [111] showed that DBSCAN outperforms traditional K-means for energy disaggregation tasks, particularly for identifying irregular appliance usage patterns that do not conform to centroid-based assumptions.

Building upon K-means foundations, hierarchical clustering combined with symbolic representation has proven superior for complex building operation analysis, outperforming traditional methods for energy signature analysis and building performance evaluation [112]. Habib and Zucker demonstrated that hierarchical clustering with symbolic aggregate approximation (SAX) and bag-of-words representation successfully identifies chiller operation patterns and enables multi-timescale energy schedule development, achieving 2–3 distinct clusters per temporal resolution [113]. Gaussian Mixture Models (GMMs) provide probabilistic clustering capabilities particularly valuable for understanding temporal pattern evolution. Advanced research [114] applied GMM with Bayesian information criterion optimization to analyze energy consumption patterns, achieving >90% pattern recognition accuracy while providing uncertainty quantification for building energy behavior analysis.

Complementing clustering methodologies, Principal Component Analysis (PCA) and dimensionality reduction techniques serve as crucial preprocessing steps for building sensor data, achieving 77% data reduction while maintaining object detection accuracy for edge computing applications [103]. The combination of PCA with K-medoid clustering enables multi-timescale schedule matrix development, allowing building systems to operate efficiently across different time scales from hourly to seasonal patterns. OPTICS clustering effectively handles varying density patterns in building systems. The latest studies [115] have demonstrated that OPTICS clustering outperforms traditional methods for fan coil unit fault detection, successfully identifying equipment anomalies without requiring pre-specified cluster numbers.

Extending beyond traditional clustering approaches, advanced clustering methods including K-shape clustering have demonstrated superior performance for time series energy data, outperforming traditional K-means with Euclidean distance and Dynamic Time Warping (DTW) approaches [116]. The normalized cross-correlation algorithm enables shape-based pattern recognition, allowing building systems to identify similar energy consumption patterns across different time periods and building zones. Isolation Forest algorithms combined with entropy weighting show remarkable performance for electricity anomaly detection. A comprehensive analysis [68] achieved a precision of 0.85 and a recall of 0.92 using enhanced Isolation Forest for abnormal consumption identification, demonstrating effectiveness for the imbalanced anomaly detection scenarios common in building fault detection. The advanced autoencoder architecture represents the current frontier in unsupervised building applications. State-of-the-art implementations [117] using Variational Autoencoders (VAE) and LSTM autoencoders consistently achieve F1-scores of 0.92–0.998 for energy consumption anomaly detection, with superior transfer learning capabilities across different building types.

These unsupervised learning approaches demonstrate a clear evolution from basic clustering to sophisticated pattern recognition methods. The following comprehensive comparison evaluates foundational machine learning approaches across multiple dimensions including accuracy, energy savings, dataset characteristics, and evaluation methodologies. This analysis addresses critical gaps in standardized performance comparison by providing detailed information about training conditions, building types, and experimental setups that enable the fair assessment of algorithmic performance across diverse smart building applications.

### 4.2. Advanced AI for Adaptive Control

While foundational machine learning techniques offer insights into building management, they often face limitations in adapting to dynamic conditions without extensive labeled datasets [118]. Additionally, traditional optimization struggles with balancing conflicting objectives, such as energy efficiency and occupant comfort [119]. To address these limitations, advanced reinforcement learning (RL) methods enable continuous learning and multi-objective optimization under uncertainty [120]. This section explores the evolution from basic RL to deep and multi-objective RL, and their integration with digital twin technology for adaptive control in smart buildings [35].

#### 4.2.1. Reinforcement Learning for Adaptive Control

**Figure 4:**
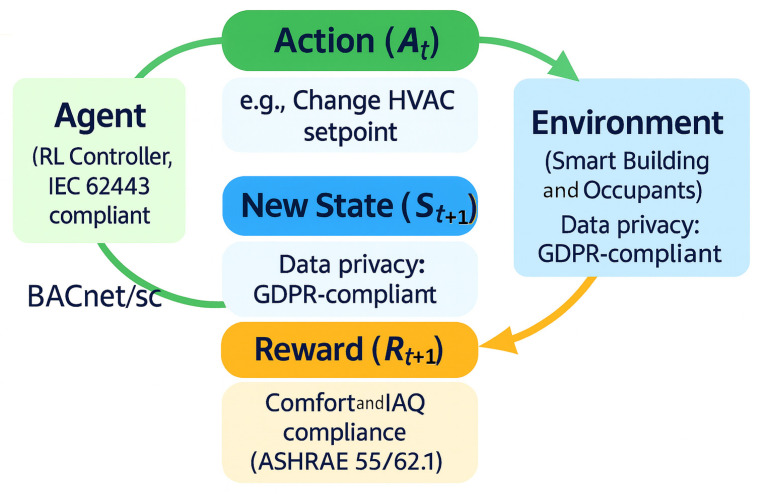
Reinforcement learning concept for smart building control.

Reinforcement learning (RL) has emerged as a powerful model-free approach for the adaptive control of smart buildings, allowing control policies to learn and self-tune through experience rather than relying on fixed rules or detailed physical models. The core RL workflow is illustrated in Figure 4, where the agent continuously interacts with the environment and adapts its control strategy based on received feedback.

In this work, the RL formulation is explicitly anchored to practical building standards. Thermal comfort is enforced per ASHRAE 55, using the canonical PMV/PPD framework and its acceptable region around neutral comfort; this turns comfort into a hard constraint during occupied hours, while energy is minimized as the primary objective [121]. For indoor air quality, we follow ASHRAE 62.1’s ventilation logic and treat CO_2_ only as a diagnostic proxy for demand-controlled ventilation (DCV), not as a prescriptive IAQ limit [122]. The reward therefore penalizes energy and demand spikes while incentivizing time within the ventilation-adequate regime. This “standards-aware” design reflects recent RL/HVAC reviews, which report best results when comfort/IAQ targets are tied to established metrics rather than ad hoc thresholds [123]. In practice, we monitor PMV near neutrality (≈−0.5…+0.5) and report the share of occupied time within the ASHRAE 55 acceptable zone, together with ventilation compliance time, as per ASHRAE 62.1.

One of the key challenges in the field of intelligent building management is the absence of standardized evaluation methods, which complicates the comparison of results from different studies. As noted in [124], “publications often lack comparability… use cases and key performance indicators vary strongly.”

In response to this challenge, the research community has proposed the adoption of unified evaluation protocols. Such protocols typically regulate a strict set of metrics for regression tasks (MAE, RMSE), classification tasks (F1, AUROC), and control tasks (RL/MORL). For control tasks, special emphasis is placed on a comprehensive analysis that includes both energy-related indicators (annual consumption, peak demand, and CO_2_e) and occupant comfort metrics (compliance with the ASHRAE 55 standard) [125,126]. Furthermore, to ensure the reproducibility of research, requirements are set for standardizing experimental conditions, such as using fixed datasets, declaring random seeds, and conducting multiple model runs.

A significant impediment to the advancement and practical deployment of artificial intelligence in smart buildings is the scarcity of standardized, high-fidelity benchmark datasets. The absence of such resources complicates the objective comparison of different solutions and hinders reproducibility. To address this gap and support the broader initiative for open science within the field, a curated selection of publicly available datasets is provided, which are appropriate for the training and evaluation of machine learning models targeting energy management, indoor environmental quality (IEQ) optimization, and occupancy sensing. The provision of these datasets establishes a crucial foundation for reproducible research and enables the equitable comparison of various algorithmic approaches. Table 6 provides a comparative analysis of these recommended datasets, detailing key characteristics such as the measured parameters, temporal resolution, and access links.

Table 7 provides a consolidated overview of the primary evaluation metrics used across the different AI task domains discussed in this review. By defining the key performance indicators for tasks ranging from energy forecasting and comfort prediction to fault detection and reinforcement learning control, this summary serves as a benchmark for comparing the performance of various models. Standardizing the understanding of these metrics is a crucial step toward ensuring the fair comparison and reproducibility of research findings in the field of smart building management.

Early RL studies demonstrated successful HVAC optimization using basic Q-learning [137], but more significant progress came from implementations that moved beyond simulation to real buildings. Deep reinforcement learning (DRL) further enhanced these capabilities, allowing neural networks to learn temperature control directly from data. Such DRL agents can continuously adapt to changing occupancy patterns, weather, and internal loads, in contrast to conventional rule-based controllers that often require manual returning [138]. Various DRL algorithms, such as Deep Q-Network (DQN), Proximal Policy Optimization (PPO), and Deep Deterministic Policy Gradient (DDPG), have been used to control HVAC setpoints, ventilation, and lighting. These agents optimize both energy use and comfort via shaped rewards. However, a critical, often understated aspect of these successes lies in the careful design of these reward functions, as poorly crafted rewards can introduce significant trade-offs, leading to unintended behavior or sub-optimal performance. Studies report 15–30% energy savings while maintaining comfort [139,140]. In [140], an RL agent saved 26% energy in a simulation and 9% in a real house compared with baseline controllers.

RL controllers excel by adapting on the fly—adjusting to unexpected occupancy changes or equipment faults—which boosts robustness in dynamic real-world environments. Field demonstrations have underscored this adaptivity: Zhang and Lam (2018) deployed a DRL policy for radiant heating in an office, marking one of the first real building RL implementations; their controller learned from a calibrated EnergyPlus simulation and successfully maintained comfort in the live system, validating RL’s practicality beyond simulation [141]. More recently, researchers implemented a deep RL (Soft Actor-Critic) agent in a modern office building’s HVAC (thermally activated slab system). After pre-training in a simulation, the agent was deployed for a cooling season and improved indoor temperature stability by 68% relative to the best rule-based strategy, without increasing energy usage—a striking real-world result of adaptive learning control [142].

These successful deployments reveal practical considerations that influence how we implement these systems. The long training periods needed for agents to perform steadily in live buildings can create operational challenges. As a result, researchers are relying more on simulation-based pre-training along with transfer learning techniques to reduce on-site learning time [138]. Safety and understanding issues have also led to the creation of guided learning frameworks and expert-led training methods. Xu et al. (2025) reported eight times faster convergence when they included domain expertise in the learning process [143].

#### 4.2.2. Deep Reinforcement Learning for Adaptive Control

**Figure 5:**
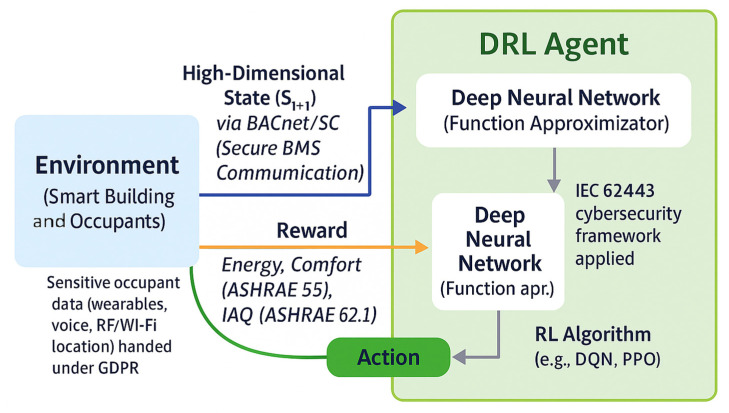
Deep reinforcement learning framework for smart building control.

Deep reinforcement learning (DRL) combines reinforcement learning with deep neural networks to overcome the scalability limitations of classical RL, enabling control in high-dimensional and complex building environments. By using neural function approximators, DRL agents can learn directly from raw sensor inputs and handle large state/action spaces that would be intractable for tabular or linear RL methods [35,143]. A landmark example is the Deep Q-Network (DQN) algorithm, which introduced techniques like experience replay and target networks to stabilize learning [144]. These innovations allowed DRL to achieve human-level control on Atari games in 2015 [144], proving the efficacy of deep neural networks in decision-making tasks. A general framework of deep reinforcement learning applied to smart buildings is presented in Figure 5.

The agent–environment exchange is implemented via the building automation system using BACnet; when deployed on IT-managed networks we adopt BACnet/SC (Secure Connect) to provide secure, IT-friendly connectivity [145], and we model the BAS network per IEC 62443 with zones-and-conduits segmentation and least-privilege conduits to the control network [146]. Contemporary BAS cybersecurity reviews highlight protocol-level weaknesses in legacy BAS stacks (including BACnet) and recommend secure variants together with defense-in-depth architectures aligned with IEC 62443 for modern, cloud-connected smart buildings—hence our choice to make the communication layer standards aware as well [144].

Building on this foundation, a variety of DRL algorithms have been developed and applied to smart building control—from value-based methods (DQN and its extensions such as Double DQN and Dueling DQN) to policy gradient methods (Deep Deterministic Policy Gradient, Proximal Policy Optimization, and Soft Actor-Critic variants) that support continuous action control [147,148]. Unlike model-predictive controllers that require explicit building thermal models, DRL agents can learn optimal control policies in a data-driven manner by interacting with simulations or real buildings [149]. This model-free approach is well suited to buildings where system dynamics are complex or poorly understood, as the agent iteratively improves its policy based on feedback rather than relying on a fixed physics-based model.

The practical implementation of these algorithms has yielded promising results. DRL applications in HVAC and energy management have demonstrated significant improvements over conventional controls. Wei et al. (2017) trained a DQN-based HVAC controller in EnergyPlus, achieving 20–70% cost reductions via a pre-cooling strategy [149]. Later studies applied DRL to multi-zone climate, lighting, and whole-building optimization. Zhang et al. (2019) cut heating demand by 16.7% using deep Q-learning in a real office [150]. These implementations typically report 10–35% energy savings while maintaining or improving comfort [151,152]. Gao et al. (2020) used DDPG to improve thermal comfort by 13.6% and reduce energy costs by 4.3% [153]. DRL enables continuous HVAC control without system simplification, balancing multiple goals via shaped rewards and showing improvements over thermostatic, rule-based, and early RL methods.

Building on these empirical successes, researchers have developed advanced DRL architectures—such as hierarchical and multi-agent frameworks—to enhance adaptive control in complex buildings by coordinating actions across timescales and subsystems, including HVAC and ventilation [153]. Recurrent neural networks (RNNs) help forecast loads and weather, improving convergence and stability [154], while attention mechanisms focus learning on relevant zones and sensors [155]. However, DRL deployment faces practical challenges. Training requires massive simulation data, and convergence is slow in large state–action spaces—millions of steps may be needed for HVAC agents. Deep networks also act as “black boxes,” reducing interpretability and trust. Recent research has explored explainable RL—techniques that provide interpretable explanations for agent decisions and policy behaviors—and safety mechanisms to address these concerns [149,156].

#### 4.2.3. Multi-Objective Reinforcement Learning

**Figure 6:**
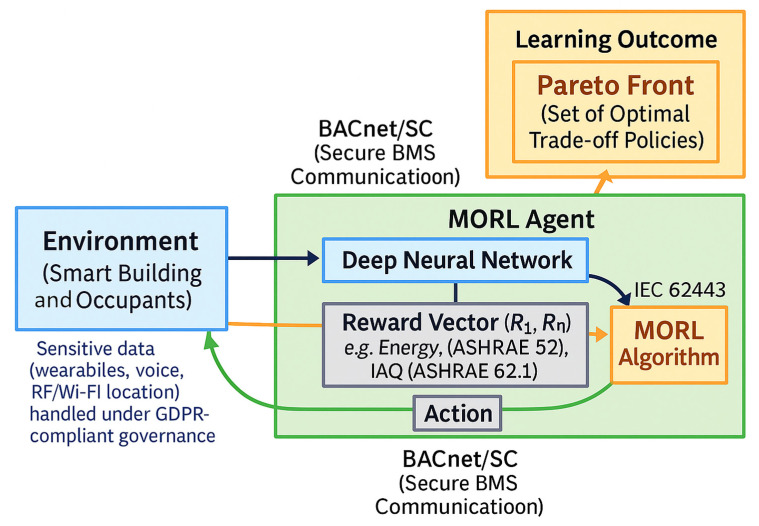
Multi-objective reinforcement learning concept for smart building control.

Smart building control inherently involves multiple competing objectives—chiefly energy efficiency versus occupant comfort—but also factors like cost, peak demand, and indoor air quality. Multi-objective reinforcement learning (MORL) extends RL to handle such trade-offs explicitly. The standard approach in building applications is to formulate a single reward that combines multiple terms (negative energy use and positive comfort) with tunable weights. This weighted sum method has been effective but requires careful tuning and lacks transparency in how each objective is prioritized. The general principle of multi-objective reinforcement learning is illustrated in Figure 6 highlighting the trade-off between competing objectives such as comfort and energy efficiency.

For multi-objective control we define a reward vector [comfort, IAQ, energy, demand][comfort,\IAQ,\energy,\demand][comfort, IAQ, energy, demand] where comfort compliance (occupied hours within the ASHRAE 55 acceptable region) and ventilation adequacy (per ASHRAE 62.1’s DCV/VRP logic, with CO_2_ as an operational proxy) are tracked alongside energy and peak demand penalties [157,158]. We report Pareto behavior (or a constrained MORL variant that guarantees comfort) because recent studies show that thermal comfort, IAQ, and energy are the dominant, often-conflicting targets in building optimization; explicitly vectorizing them yields transparent trade-offs and reproducible KPIs [159,160].

Brandi et al. (2020) trained a deep RL agent with a weighted reward to reduce heating energy while maintaining comfort, though the weight tuning required adjustment in cold conditions [161]. Balancing objectives remains challenging: emphasizing energy can cause discomfort, while prioritizing comfort increases energy use. Researchers often dynamically adjust weights to reflect changing priorities. Advanced MORL methods learn policies that handle multiple objectives or offer Pareto-optimal trade-offs. A review of 74 studies (2010–2025) found that RL controllers often save >20% energy while maintaining comfort by adjusting reward weights [162]. Some methods maintain separate value functions per objective to select non-dominated actions. Authors in [163] proposed proposed a human-centric DRL agent optimizing energy, comfort, and air quality. It achieved 8% energy savings in focused mode and improved comfort by up to 21% when prioritized [164].

Another promising direction is treating comfort requirements as constraints rather than penalties. Recent work has introduced safety constraint algorithms and two-tier control schemes to ensure comfort is never violated while still minimizing energy. One such approach involves applying a runtime “shielding” layer that adjusts the HVAC actions of a trained RL agent to enforce temperature bounds, instead of relying on a large comfort penalty in the reward [143]. This kind of approach effectively turns the multi-objective problem into a constrained optimization (maintain comfort, minimize energy), and early results show it can eliminate thermal discomfort events without sacrificing much efficiency. Multi-objective formulations have also been extended to consider occupant preferences and grid-level objectives. Lei et al. (2022) demonstrated an occupant-centric DRL controller that simultaneously managed temperature, CO_2_, and lighting in multiple zones; by learning a policy that responds to occupant feedback (preferences) as well as sensor data, it improved the overall comfort index while still saving energy across a variety of operating scenarios [165]. Such occupant-centric MORL reflects the real-world need to juggle individual satisfaction against aggregate energy goals in smart buildings.

When occupancy and comfort estimation rely on wearables, voice, RF/Wi-Fi location, or camera-based analytics, our data governance follows GDPR-aligned principles—lawfulness and transparency, purpose limitation to building control, and data minimization with strict retention/pseudonymization [166]. Large-scale user studies in smart commercial buildings show that occupants have heterogeneous privacy preferences and often lack an awareness of data collection [167]; practical deployments should therefore provide clear notices and opt-in mechanisms while limiting high-inference modalities unless strictly necessary for control. Our pipeline implements these safeguards and audits inference risk for each sensing modality before activation.

Modern MORL research combines improved algorithms and broader objective modeling. Key techniques include Pareto Q-learning, preference-weighted policy gradients, and decomposition-based learning. Applications now cover objectives such as demand response and indoor environmental quality.

#### 4.2.4. Digital Twin Synergy with DRL and MORL

**Figure 7:**
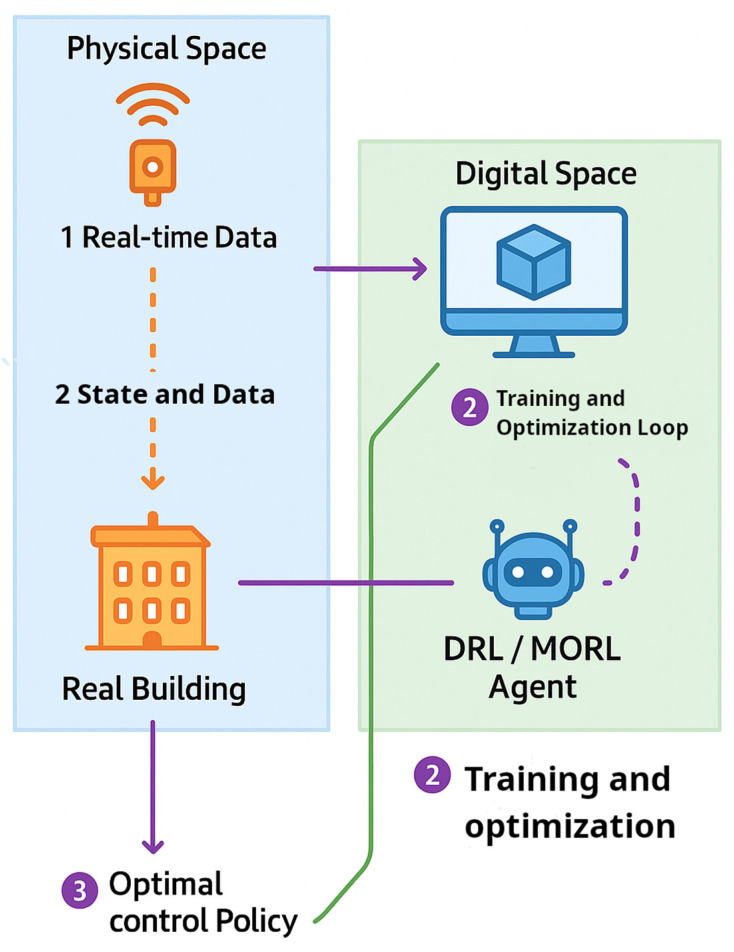
Integration of digital twin with deep and multi-objective reinforcement learning.

Digital twins—virtual replicas of real buildings—enable the safe training and deployment of DRL and MORL agents by reducing sim-to-real gaps. Silvestri et al. (2024) showed that a twin of an office HVAC system, trained with historical data, allowed an SAC agent to reduce peak temperature deviations by ~68% after real deployment without increasing energy use [108]. Beyond training, twins support scenario-based optimization—a process where control algorithms are tested against multiple operational scenarios including varying weather patterns, equipment failures, peak occupancy events, and grid outages to identify robust control strategies before real-world deployment [167]. This approach enables MORL agents to systematically explore diverse conditions and generate Pareto-efficient strategies that balance comfort and energy performance across different operational contexts [163]. The twin provides a simulation sandbox for testing and tuning policies under these varied scenarios [168]. Figure 7 shows how digital twins interface with DRL and MORL agents to support simulation-based training, scenario testing, and safe deployment in real buildings.

**Figure 8:**
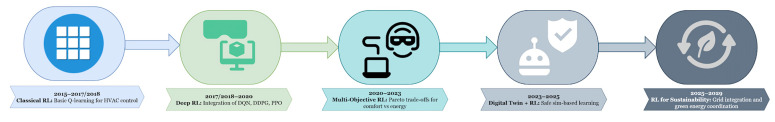
Stagewise evolution of AI methods in smart building control.

Digital twins can also function in real time by syncing with IoT sensors and supporting closed-loop control. RL agents can test actions virtually before live deployment. Spudys et al. (2023) used a campus twin to predict performance tiers, showing how such systems support safe decision-making [139]. In advanced setups, the twin becomes part of the control loop (virtual commissioning), enabling online adaptation to building changes. Veje and Jørgensen (2021) demonstrated a twin-based framework that used feedback and data to co-optimize energy and comfort [167]. These examples show how twins enable interpretable, multi-objective control through simulation. The chronological development of AI techniques applied in smart building control is summarized in Figure 8 showing the transition from classical reinforcement learning to deep, multi-objective, and sustainability-focused approaches, including integration with digital twins.

Digital twins face deployment barriers. Model creation and calibration are time-consuming, and live twins require costly infrastructure and data pipelines [168]. Kannari et al. (2025) note that most AI + twin integrations remain untested in real buildings due to data silos and system fragmentation [169]. With growing IoT adoption, twin–AI convergence is accelerating. Prototypes from Siemens, Bosch, and lab studies show twins enabling closed-loop RL deployment while reducing commissioning time [170].

The integration of digital twins fundamentally transforms the deployment characteristics of different AI-based control approaches. Classical reinforcement learning benefits from digital twin simulation environments for safer training, achieving moderate energy savings of 10–20% with straightforward implementation, though it is limited to handling 5–15 state variables [140,142]. Digital twin pre-training reduces the risk of sub-optimal control during real-world learning phases.

Multi-objective reinforcement learning extends optimization capabilities to balance competing objectives simultaneously, achieving 20–30% energy cost reductions while maintaining comfort constraints [140,166]. The tunable DQN approach demonstrated 30% cost improvements with 18.2% peak demand reduction [157], though computational complexity increases exponentially with additional objectives. Digital twin integration represents the most sophisticated approach, combining physics-based modeling with RL agents to achieve 15–25% energy savings while providing superior interpretability through virtual building representations [145]. Pre-training in digital environments accelerates convergence by 60% compared with direct real building learning, though implementation requires 24–36 weeks’ development and substantial infrastructure investment.

State space handling capabilities distinguish the approaches significantly: classical RL manages discrete low-dimensional problems, DRL processes 20–100+ variables through neural function approximation, MORL handles 30–200+ variables with multi-objective coordination, and digital twin systems accommodate 100–1000+ variables through integrated physics modeling [35]. Real-time deployment readiness varies from easy implementation for classical RL to complex integration requirements for DRL and MORL, while digital twin systems achieve medium complexity after the initial setup phases. To summarize these advanced AI methods for smart building control, Table 8 provides a clear, side-by-side comparison of their key characteristics, helping to illustrate their individual strengths and applications.

### 4.3. Generative AI and Language Models for Comfort and Health Management

The evolution of smart buildings has predominantly been centered on prediction and reaction strategies. A new shift is taking place due to generative artificial intelligence (AI) and, in particular, large language models (LLMs). Unlike previous models that relied on simple data pattern recognition, generative models go far beyond reasoning, creating, and communicating in uniquely human ways, fully transforming environments and smart building interaction [171]. An important phenomenon called “emergent abilities” has been documented, which reflects the emergence of multi-step reasoning as models become larger, where smaller versions of the model did not demonstrate such features [172]. Because of this enhanced sophistication, LLMs such as the GPT models are more powerful. They are capable of comprehending the nuances and intents of human expressions which make truly intelligent interactions between humans and spaces possible. This chapter discusses the ways advanced AI moves beyond automation and shifts towards anticipatory systems for personalized comfort, health, proactive care, and sophisticated operations to finally allow natural language to be the primary interface user command tool for the built environment.

#### 4.3.1. Natural Language as the New User Interface

**Figure 9:**
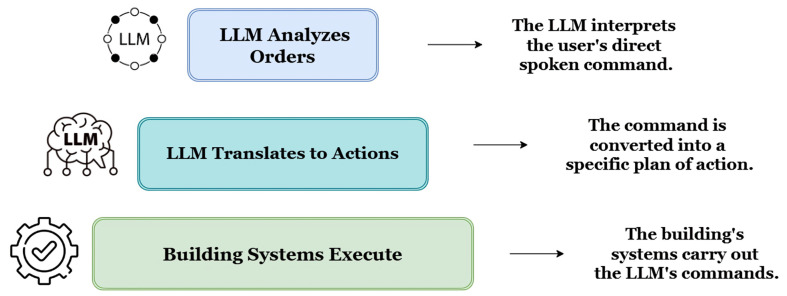
AI-driven environmental adjustment.

LLMs are changing smart buildings by removing strict interfaces. For many years, buildings interacted with users through limited apps or old thermostats. Now, they can communicate using speech as shown in Figure 9. With LLMs, natural language becomes a smooth interface, allowing people to share their feelings like saying, “it feels a bit gloomy in here” and having an AI agent turn that into actions such as adjusting lights or raising blinds [173]. This goes beyond simple voice control; it focuses on grasping the intent behind commands. While LLMs have shown skill in HVAC systems by passing certification exams, their real value is not just repeating information [174]. Their current limitation lies in applying that knowledge consistently; they often struggle with calculations and ambiguous, open-ended problems common in real life [175].

Despite these challenges, progress is clear. In labs, assistants like DAVE can understand spoken commands to alter complex Building Information Models (BIMs) with about 94% accuracy for simple tasks [176]. The trouble arises with complexity; DAVE’s success rate drops to just under 50% when handling multi-step requests. This highlights the difficulty these models have in understanding context and following sequences. Yet, researchers are focused on more than just managing a building’s physical environment. They are using LLMs to create immersive, interactive spaces, such as a simulated traffic environment that helps autistic children learn social cues safely [177]. This marks a significant shift from managing physical comfort to supporting psychological and social well-being, imagining a future where buildings serve as empathetic partners. However, this hopeful vision is challenged by a significant issue: LLMs can “hallucinate,” or create false information. A 6% hallucination rate may seem minor in some cases, but in a safety-critical system or educational tool for vulnerable users, it is unacceptable. This points to an urgent need for thorough validation, fail-safe mechanisms, and human oversight to ensure that every AI-driven action is safe and accurate [178,179].

#### 4.3.2. Hyper-Personalization Through Fine-Tuning and Adaptation

If natural language is the new interface, then hyper-personalization is the new experience. The real power of generative AI lies not just in understanding general commands, but in learning to understand individual users. Foundational LLMs are trained on a vast amount of anonymous internet data, but they can be fine-tuned to focus on the unique information of an individual. By using someone’s biometric data from a wearable, their calendar, their written feedback, or even their specific way of speaking, a general model can become a personal aide for well-being [180,181]. This is the essence of personalized medicine, where treatment is tailored to a person’s unique biological and lifestyle data [182,183]. To be effective, this process must extend beyond data, accounting for local customs and cultural nuances, whether in a diverse healthcare setting or a global company where comfort can mean different things [184].

The impact on health could be significant. Imagine a building that does not just react to a fever but helps prevent one. Generative models can gather information from wearables to manage health proactively. Frameworks like PhysioLLM and Health-LLM are being developed for this purpose, while the COMFORT framework uses ongoing, efficient fine-tuning to create early-stage disease detectors that can run on edge devices, cutting memory requirements by half [185,186]. LLMs are already being used to create customized exercise plans and patient care strategies, freeing up time for clinicians and potentially enhancing safety by providing advice tailored to a person’s specific health profile [187,188]. However, this deep level of personalization raises important concerns. It involves access to sensitive data, creating significant privacy and security risks. Furthermore, these models can pick up and amplify societal biases present in their training data, possibly leading to worse outcomes for already-marginalized groups [189,190]. Their “black box” nature and lack of responsibility for mistakes are also major barriers to use in high-stakes areas like medicine, where errors can have serious consequences, and clinicians need to understand the reasoning behind recommendations [191]. Additionally, the models lack true emotional intelligence, which is critical for interactions involving human vulnerability [186]. In response, researchers are advocating for more transparency with initiatives like the TRIPOD-LLM reporting guidelines, which require researchers to clarify their data, methods, and the limitations of their models [192].

#### 4.3.3. Enhancing Building Operations with Generative Models

While much of the excitement about generative AI centers on the experience of occupants, its impact on building operations is equally groundbreaking. One of the biggest challenges in building management is the shortage of reliable data, especially for rare but important events like equipment failures. Generative models, such as GANs and VAEs, can serve as a “digital sparring partner” for other AI systems, generating large amounts of realistic, synthetic sensor data to train on [193,194]. This enables developers to simulate a variety of scenarios, including equipment breakdowns, to create stronger predictive maintenance and energy forecasting models. This might result in their application in medicine, where synthetic patient data is used to train diagnostic models without compromising privacy, which is an approach relevant for protecting sensitive occupants or operational data in a corporate environment.

LLMs are also set to become invaluable resources for facility managers. Currently, a fault detection system might present a confusing error code. Soon, an LLM could review that data and give a clear report in natural language: “Heads up, the energy use for Air Handler 3 is up 20% this week, and airflow is down. It is likely a clogged filter that needs replacing” [195]. This ability to convert data into practical insights makes expertise accessible, similar to how LLMs are used in healthcare to automatically summarize patient notes and reduce doctors’ administrative burdens [191]. This significantly lowers the barrier to understanding and managing complex building systems. The potential is vast: by automatically creating building energy models from simple descriptions or supporting decisions for retrofitting, LLMs could cut modeling efforts by more than 95% [195]. However, this promise is limited by their current and significant unreliability in math- and physics-based reasoning. It is not just about making a calculation error; it reflects an underlying struggle with the symbolic logic required in engineering, making them untrustworthy for the precise calculations needed in tasks like heat load analysis or structural design [178,195].

#### 4.3.4. Cloud-to-Edge Deployment Architectures

**Figure 10:**
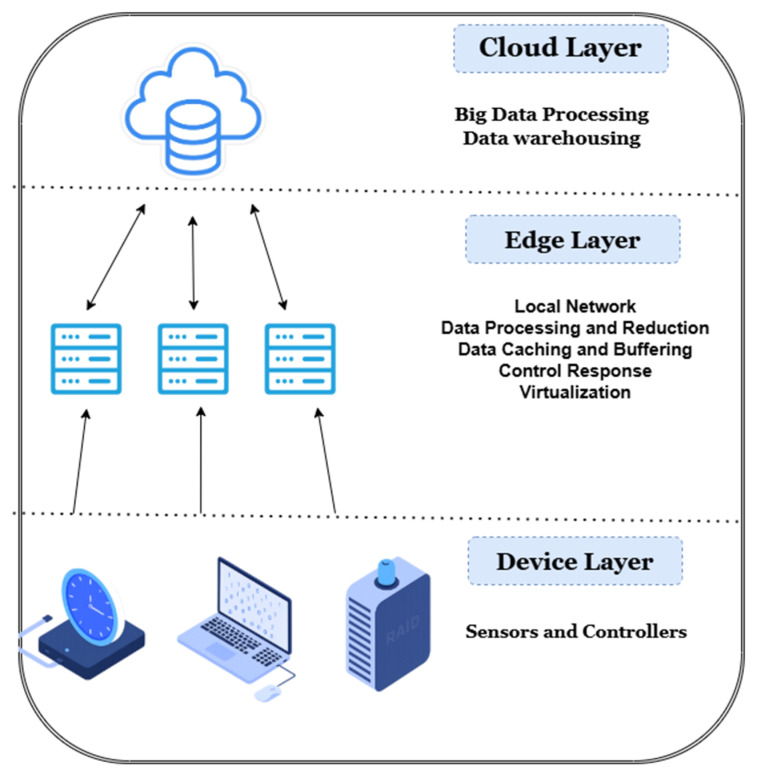
Edge–cloud architecture.

Implementing this level of intelligence in a building is not as simple as installing a new app. The intense processing power of a large language model cannot fit on a small sensor mounted on the wall. The costs, both computational and financial, are too high [177,196]. The solution is a hybrid structure that acts like a central brain with local responses: a cloud-to-edge model (Figure 10). In this arrangement, the more demanding tasks, training, fine-tuning, and complex reasoning are handled on powerful servers in the cloud [187]. This method is often viewed as the most effective for latency-sensitive, hybrid tasks, ranging from smart buildings to industrial automation [197].

For a building to react instantly, it cannot wait for data to travel to the cloud and back. That is where the local responses come into play. Smaller, specialized models are set up on “edge” devices within the building [198]. These agile models are often simplified versions of their larger cloud-based counterparts, trained to manage immediate tasks with minimal delay [187]. This distribution of work makes for a strong system: the edge model takes care of instant commands like “turn on the lights,” ensuring that the system functions even without internet access. Meanwhile, the cloud brain analyzes long-term patterns and provides new intelligence and updates. This setup can also enhance privacy, as sensitive data can be processed locally. However, crafting this decentralized system involves essential trade-offs. A federated learning method works well for privacy but can be slow and exposed to risks. A serverless architecture is cost-effective for occasional tasks but suffers from delays, making it unsuitable for continuous control [197]. The hybrid model shows the most promise, but it brings its own challenges in managing data flow, defending against a larger potential for security threats, and handling the computational demands on devices not made for this level of intelligence [197,198].

## 5. Discussion

An analysis of studies reveals a steady shift from rigid rule-based systems to adaptive environments that account for occupant comfort, health, and behavior. AI algorithms are the main driver: applying deep reinforcement learning lowers energy consumption by 10–35% without sacrificing comfort [199]. These gains are enabled by a growing array of sensors, connected devices, analytics tools, and user interfaces that form the technological foundation of smart buildings.

The transition from pilot projects to large-scale deployment is hampered by data, integration, and ethical issues. AI models require large, high-quality datasets, yet standard public datasets are scarce, complicating the objective comparison of solutions [199]. The spread of audio and video sensors increases privacy risks [200].

Data quality is closely tied to broader ethical challenges, including the reproducibility crisis. Only a small fraction of key findings can be confirmed by independent studies, casting doubt on the reliability of the underlying datasets [201]. The lack of harmonized data management practices across research and clinical centers exacerbates scarcity: quality control methods vary, making datasets hard to interpret and compare [202,203]. Data sharing is hindered by concerns over privacy, intellectual property, and the reputational risks associated with exposing data quality shortcomings [201,204].

Recent studies highlight the necessity of aligning smart building systems with frameworks like the General Data Protection Regulation (GDPR). GDPR’s “privacy by design” principle mandates data anonymization and user consent in occupant monitoring systems, emphasizing the need for granular access controls to mitigate re-identification risks in HVAC and occupancy datasets [202]. Federated learning (FL) has been proposed as a privacy-preserving approach, allowing decentralized model training without sharing raw data and thereby maintaining occupant anonymity [203]. Cross-border data transfers introduce additional complexities, with EU–US Privacy Shield alternatives evaluated post invalidation. Hybrid cloud–edge architectures are recommended to localize data processing and avoid jurisdictional conflicts [197]. Ethical AI deployment also requires explainability and bias mitigation. Explainable AI (XAI) tools like SHAP and LIME can audit black box models in building automation, ensuring transparency for stakeholders [205]. Integrating XAI with BIM allows occupants to query and understand AI-driven HVAC adjustments, providing transparency in automated decision-making [206]. Synthetic data generation, for instance via GANs, addresses data scarcity while adhering to privacy laws. Synthetic occupancy patterns can retain statistical fidelity without exposing real identities [207].

The following approaches mitigate these risks. Federated learning trains models on distributed devices without transferring sensitive information [207]. Synthetic datasets generated with GANs and variational auto-encoders enlarge training corpora without using real occupant records [207]. Blockchain provides secure, auditable data exchange between stakeholders [208]. Unified data models and ontologies enhance system interoperability and simplify information interpretation across platforms [209].

One of the primary cybersecurity risks linked to fine-tuning is the heightened susceptibility to adversarial attacks. Because fine-tuning can erode the robustness of the original pre-trained model, attackers may exploit these weaknesses by injecting minor perturbations into inputs that force the model to misclassify or generate harmful outputs. Adversaries can engineer inputs that cause a machine learning-based intrusion detection system to ignore malicious activity or, conversely, to flood it with false alerts that overwhelm security operations [210]. Deploying fine-tuned models in mission-critical cybersecurity applications without strong adversarial defenses can lead to major breaches, data leaks, and operational disruptions [210,211].

One of the key technical challenges lies in integrating modern AI systems with the existing infrastructure in buildings. Most current automation systems were not designed with openness or compatibility in mind, which makes integration costly and technically demanding [206]. Digital twins (DTs) have become a promising solution. By creating virtual replicas of real-world buildings, DTs allow researchers and engineers to simulate AI-driven management strategies safely before deploying them in actual environments [212]. These digital models can merge data from multiple sources—such as Building Automation Systems (BAS), IoT devices, and weather forecasts—into one framework. However, building and maintaining these models is resource-intensive and becomes more difficult when scaling across many buildings [213]. Research now points to the value of standard frameworks and open-source platforms, which could simplify development and reduce compatibility barriers. Using widely accepted communication protocols like BACnet/IP, Modbus TCP, or newer ones like Matter also helps unify devices from different vendors [214]. Middleware tools and API gateways are essential for bridging the gap between legacy systems and modern AI-driven platforms.

As AI tools grow more complex, understanding how they make decisions becomes more difficult, especially with methods like DRL and large-scale language models. This lack of transparency, sometimes called the “black box” issue, makes it harder for stakeholders to trust the system [208]. An AI system acting autonomously could make decisions that are not immediately understandable, raising concerns about comfort, safety, or operational reliability.

To counter this, explainable AI (XAI) methods are gaining traction. Tools like SHAP and LIME help break down model predictions and offer insights into why a particular decision was made [209]. Incorporating human-in-the-loop (HITL) approaches is also important—these combine AI automation with oversight from human operators who can intervene or validate outcomes when needed [214]. Frameworks for testing and verifying AI systems are essential to ensure they meet safety and performance requirements before being deployed [215]. On a broader scale, legal and ethical guidelines tailored to AI use in buildings are needed to ensure responsible deployment and increase public acceptance [216].

Future developments must blend engineering, policy, and user-focused design. Successful smart building systems should be open, secure, and easy to use, while always centering the needs of the people living and working inside them. Future research should explore scalable architectures, fair data governance policies, and technologies that are both adaptable and explainable. Continued collaboration between researchers, developers, policymakers, and occupants will be essential to ensure that AI in smart buildings improves quality of life without introducing new risks.

**Figure 11:**
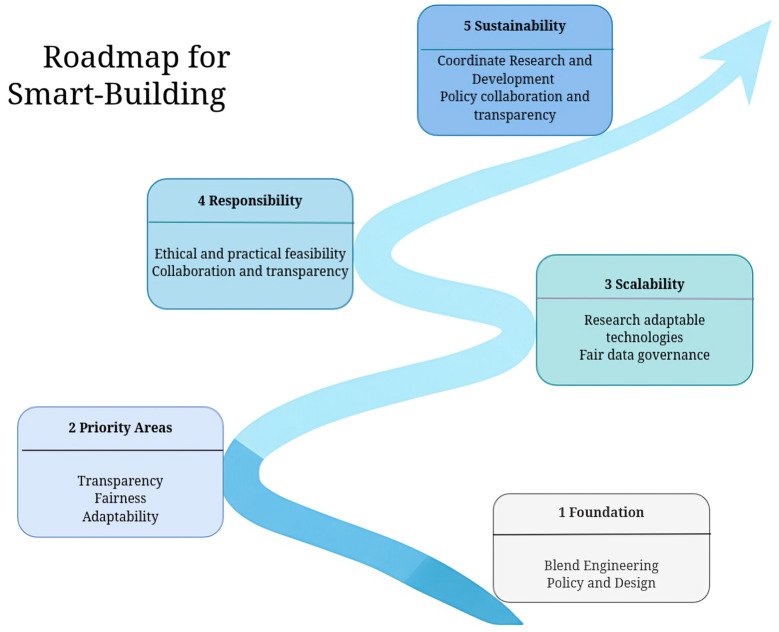
Roadmap for smart building AI.

A forward-looking roadmap is required to steer innovation in smart building AI while maintaining ethical responsibility and practical feasibility. Recent studies converge on staged frameworks that coordinate research, development, and policy over clearly defined short- and mid-term windows. These frameworks extend beyond technical targets to foreground transparency, trust, security, and sustainability. Figure 11 distills the priority areas and concrete action steps for the next five years, outlining a clear path toward responsible and scalable smart building systems.

Large-scale smart buildings deploy extensive networks of sensing devices to monitor environmental and operational parameters, yet the size and complexity of these networks create significant maintenance and optimization challenges. Thermoelectric Generators (TEGs) have shown significant potential for powering autonomous sensors by converting thermal gradients into electricity, which reduces maintenance requirements and enhances sustainability [50]. Similarly, optimal wireless sensor network (WSN) topologies can minimize energy consumption and improve stability through distributed control, addressing scalability challenges in IoT-enabled buildings [40]. Accurate device localization and optimization in large buildings remain complex due to heterogeneous sensor densities and dynamic environmental conditions. Integrating edge computing with Named Data Networking (NDN) can reduce latency and improve reliability in data retrieval, enabling real-time sensor coordination [43]. Decentralized machine learning frameworks further enhance privacy and efficiency in cloud–edge architectures, which is crucial for managing distributed sensor networks [42]. Autonomous optimization is another critical focus. Continuous calibration frameworks automate the synchronization of a building’s digital twin with its physical counterpart by dynamically adjusting model parameters in real time, ensuring a high accuracy with minimal human intervention. The system continuously ingests sensor data and uses a pre-trained model to estimate unobservable variables like occupant count or equipment heat, updating the digital twin instantly. A real-time validation loop compares the model with live data to maintain precision, while the automated denoising and handling of missing data enhance resilience. This enables the digital twin to self-correct, adapt to changing conditions, and support proactive decisions [71]. AI-driven surrogate models can predict airflow patterns, optimizing HVAC sensor placement and reducing computational overhead [72]. Energy harvesting solutions integrated with Building Information Models (BIMs) support intelligent energy management, reducing the dependency on external power sources [39].

Privacy-preserving techniques such as federated learning and blockchain help mitigate security risks while ensuring scalable sensor networks [207]. Hybrid cloud–edge architectures automate the distribution of computational tasks, with edge devices handling lightweight processing and cloud servers managing intensive computations. This setup enables real-time autonomous optimization, where local models are continuously trained and updates aggregated to refine the global AI model. Automated feedback loops allow the system to adapt quickly to changing conditions without human intervention. Privacy and data integrity are maintained by keeping raw data on local devices and transmitting only anonymized model updates. This approach ensures efficient, secure, and intelligent operation across the network [197]. These advancements prioritize sustainability, scalability, and autonomy in large-scale smart building systems.

## 6. Conclusions

This review summarizes the current state of research on the application of artificial intelligence technologies for proactive comfort and health management in smart buildings. The analysis shows that the integration of IoT-based sensor networks, advanced communication protocols, and AI-driven data processing forms the foundation of a new generation of buildings capable of continuously adapting to changing environmental conditions and individual user needs. Through a multi-layered architecture—including sensing, edge and cloud computing, actuation systems, and user interfaces—modern buildings are shifting from reactive automation to proactive, human-centered ecosystems.

The review demonstrates that AI methods, particularly supervised and unsupervised machine learning, reinforcement learning, and their deep and multi-objective variations, significantly enhance the efficiency and adaptability of control strategies. Applications include occupancy and thermal comfort prediction, as well as the implementation of energy saving policies without compromising user well-being. Digital twin technology amplifies these capabilities by enabling safe training, optimization in simulation environments, and real-time closed-loop control. Studies report energy savings in the range of 15–35% while also improving indoor environmental parameters such as CO_2_ concentration, temperature stability, and air circulation.

Despite this progress, several challenges remain. Data silos, the lack of standardized evaluation methods, and privacy concerns related to personal sensing technologies hinder widespread adoption. Moreover, the complexity and resource intensity of deploying advanced AI models—especially those involving deep learning and hybrid digital twins—require substantial investment and interdisciplinary collaboration. The need for explainable AI, privacy-preserving algorithms, and ethical principles is growing as buildings evolve into cyber–physical systems that handle increasingly sensitive user data. Future research should focus on the development of modular and interoperable platforms that allow integration across different building systems and equipment vendors. Equally important is the publication of open datasets and the creation of simulation environments that ensure the reproducibility and comparability of results. Personalized control strategies, comfort modeling using wearable devices, and self-adaptive intelligent agents are key directions to bridge the gap between technical feasibility and practical usability.

## Figures and Tables

**Table 1:** Consolidated data extraction across studies.

Building type	Climate	Sensors	Algorithms	Datasets	Baselines	Metrics	Validation methods	Scenario	Cost–benefit analysis	
Smart city/industrial IoT/campus	Heterogeneous	Generic IoT (implied)	NSGA-II; stochastic programming	Synthetic (IoT traffic simulation)	IP-centric arch.; equal weights	Latency, throughput, energy, cache hit, reliability, PDR	Testbed, simulation, sensitivity, standards comparison	Simulation + testbed	−25% latency; +15% energy eff.; +20% cache; 95% reliability w/30% edge failures; trade-offs	[19]
Residential/commercial/healthcare	Not reported	IoT sensor networks; smart meters (elec/water/gas)	FL, edge, ML (NN, SVM), SITA	Smart meter data (discussed)	Binary privacy settings	IAQ prediction accuracy; system perf (privacy-preserving)	Concept + systematic review	BMS/IoT integration (platform)	↑ privacy/compliance; ↓ risk (edge); faster monitoring/control	[20]
Offices (university)	Not reported	Illuminance, occupancy, temperature (IoT)	ABM (AnyLogic); EnergyPlus	Weather, floor plan, occupancy profiles	Traditional schedules; manual behavior	kWh (total/lighting/HVAC), comfort	10 min step × 365-day simulation; demo case	Simulation	−31.71% total; for wasteful behavior: lighting −60%, HVAC −31.47%, total −35.52%	[21]
Kindergartens (classrooms)	Seoul, January–July 2022	PM2.5/PM10/CO_2_/T/RH; window sensors	Pearson’s correlation	Indoor/outdoor measurements at 10 sites	KR 25; ASHRAE 18 m^3^/h·person	PM, CO_2_, T/RH, window open time, ventilation per person	Python analysis; comparison to standards; CO_2_ evaluation	Field	Ventilation 1.77–10.69 < norms; window-dependent; low-cost sensors	[22]
University buildings	Zaragoza; winter/summer	CO_2_, T, RH, pressure; elec meters	Correlations; occupancy-based fan coil control	RT and long-term; SCADA; weather; PV	Design calcs; RD 1826/2009; 2022; CO_2_ 800/1000; Passivhaus 120; CTE 184	kWh, CO_2_, T/RH, standby, occupancy, comfort, BEPG	Continuous monitoring; sensor comparison; temp trends	Field	HVAC −40–70%; standby 285 MWh/yr (64% potential); occupancy opt. −53–63%; low OPEX/eco-costs	[23]
Industrial (M/P/C)	Not reported	PMU, CT	Node-RED; MQTT	Time series for chillers/boilers/HVAC/dust collectors/equipment	Not reported	U, I, P/Q/S, f, PF, kWh, data accuracy	Continuous monitoring; audit; single line; integration tests; MQTT Explorer	Field	↑ efficiency; ↓ waste; scalable; reliable low-latency LAN	[24]
Intelligent building (air-conditioned spaces)	Not reported	Electricity meters	BIM; adaptive GA; Matlab model (Matlab 24.2)	Simulation + experimental data	Conventional control	Energy savings; kWh; comfort index P; CO_2_	2-zone setup; model vs. experiment; statistics	Field + simulation	−11.43% energy at equal comfort	[25]

**Table 2:** Sensor and IoT technologies for smart building systems.

Technology	Measured Parameters	Application	Advantages	Research References
Indoor Air Quality (IAQ) Sensors	Temperature, humidity, CO_2_	IAQ monitoring in sensitive environments	Critical role in ensuring healthy conditions, providing granular health data.	[37]
Industrial IoT	Energy consumption	Optimization of energy consumption in manufacturing facilities	Real-time monitoring, optimization of energy management, waste reduction.	[38]
IoT Sensors for Building Management Systems (with BIM)	Data for optimizing equipment energy saving	Online monitoring and management of building equipment with Building Information Modeling (BIM) integration	Intelligent control, increased energy efficiency.	[39]
Wireless Sensor Networks	Data for distributed optimal control	Efficient data collection in smart buildings	Optimal communication topology design considers energy consumption and stability.	[40]

**Table 3:** Comparative analysis of sensing technologies for smart buildings.

Technology	Measured Parameters	Relevance to Comfort and Health	Strengths	Weaknesses	References
Passive Infrared (PIR)	Motion (presence)	Low: Basic detection to switch systems on/off.	Low-cost, high energy efficiency, non-invasive.	Cannot distinguish occupant count; ineffective for stationary occupants.	[51]
Cameras (CV)	Count, position, activity	High: Provides precise data for metabolic load calculation.	Maximum data granularity.	Critical privacy concerns; high-cost.	[52]
Radar (mmWave)	Count, position, respiration	High: Occupant counting and respiration rate for air quality control.	Privacy-preserving, high-accuracy, works in darkness [56].	Relatively high-cost; signal processing complexity.	[56]
Wi-Fi (CSI)	Presence, count, activity	Medium: Low-cost occupancy estimation for zonal control.	Uses existing Wi-Fi network; no extra hardware needed.	Sensitive to environmental changes; complex calibration.	[54]
Wearable Devices	HR, HRV, temperature, activity	Very High: Direct physiological data for personalization.	Direct physiological measurement, foundation for personalization [59].	Requires user to wear and charge; depends on user consent.	[57]

**Table 4:** Sensor–metric–standard mapping with example multimodal fusions and associated trade-offs.

Sensor Type	Primary Metric(s) Mapped	Standard Anchor	Typical Fusion	Key Trade-Offs	References
CO_2_ (NDIR)	IAQ/ventilation adequacy (proxy)	62.1 VRP logic; CO_2_ as a proxy indicator; elevated CO_2_ at steady state → insufficient ventilation	CO_2_ + PIR/mmWave (DCV)	low • very low • privacy-friendly • bias: not direct occupancy count	[62,63,64]
Temp/RH/Air speed	Operative T, PMV/PPD (comfort)	Comfort compliance via ASHRAE 55 acceptable zone	Temp + RH + air speed (PMV); +wearables	low • low • privacy-friendly • bias: location/calibration	[60,61]
PIR	Presence (binary) → DCV/comfort triggers	Linked to 62.1 occupancy schedules and 55 occupied hours	CO_2_ + PIR, Temp + PIR	very low • very low • high privacy • bias: static occupants	[62,65]
mmWave radar	Presence/count, micro-motion/respiration → exposure proxy	Indirect link to comfort/IAQ control targets	mmWave + CO_2_, +wearables	medium • low • very high privacy • bias: environmental clutter	[67]
Wi-Fi CSI	Presence/activity (indirect occupancy)	Indirect link to occupancy targets, supports zonal monitoring	CSI + CO_2_	very low • very low • high privacy • bias: sensitive to environmental drift	[68]
Wearables	HR, HRV, skin temperature, activity → personalized comfort	Participant-oriented comfort models	Wearables + zone sensors	device cost • charge cycle • consent required • sampling bias	[60,61]
Acoustic	dBA → ergonomics (noise)	Ergonomics (visual/acoustic) as secondary metric	dBA + PIR (occupied hours)	low • low • privacy risk if raw audio → use edge features	[65]

**Table 5:** Comparative analysis of foundational machine learning approaches for smart buildings.

ML Approach	Applications	Accuracy Range	Energy Savings	Key Advantages	Dataset	Train/Test Ratio
XGBoost	Energy prediction, comfort, fault detection	92–97% [92,93,94]	15–27% [92,93]	High accuracy, handles missing data, structured input	Multi-system HVAC data from commercial buildings (temperature, humidity, occupancy, weather)	70% training, 30% testing
Random Forest	Forecasting, occupancy detection	89–97% [88]	10–20% [88]	Interpretable, robust, feature importance ranking	Human physiological and subjective responses	80% training, 20% testing
SVM	Consumption forecasting, classification	87–91% [82]	10–15% [82]	High precision, theoretical robustness	Simulated hourly building climate and cooling load	20% training, 80% testing
K-means	Energy pattern discovery, user clustering	85–89% [95]	5–15% [95]	Simple and effective, low computational cost	Hourly/weekly energy consumption data from 10 institutional buildings	80% training, 20% testing
Hierarchical	Multi-scale operation analysis	87–92% [96]	10–18% [96]	No preset clusters needed, flexible resolution	BAS data from 247 thermal zones and one AHU	Not mentioned

**Table 6:** Summary of smart building datasets and their characteristics.

Dataset Name	Measured Parameters	Temporal Resolution	Reference
CU-BEMS, smart building energy and IAQ data	Electricity consumption (individual AC units, lighting, plug loads), indoor temperature, relative humidity, ambient light.	1 min	[62]
Smart Building System	CO_2_ concentration, humidity, temperature, luminosity, PIR motion (occupancy).	5 s (most sensors), 10 s (PIR)	[127]
AlphaBuilding	A synthetic dataset covering HVAC, lighting, miscellaneous electric loads (MELs), occupant counts, and environmental parameters.	10 min	[128]
Lawrence Berkeley National Laboratory FDD Datasets	Time series data for both normal and faulted HVAC operations (e.g., rooftop units, chillers, air handlers). Includes fault-free and various fault severity levels.	Varies by system	[129]
REFIT Electrical Load Measurements	Aggregate and appliance-level power consumption from 20 UK homes, along with occupancy and survey data.	8 s (appliance-level)	[130]

**Table 7:** Comparison of common evaluation metrics in smart building research.

Task Domain	Common Metrics	Description	Reference
Energy and Load Forecasting	RMSE (Root Mean Square Error)	Measures the standard deviation of the prediction errors. Very sensitive to large errors.	[131,132]
	MAE (Mean Absolute Error)	Measures the average magnitude of the errors in a set of predictions, without considering their direction.	
	R^2^ (R-squared)	Indicates the proportion of the variance in the dependent variable that is predictable from the independent variable(s). A value closer to 1 indicates a better fit.	
	CV-RMSE (Coefficient of Variation in the RMSE)	Normalizes the RMSE to the mean of the measured values, allowing for comparison across datasets with different scales.	
Comfort and Occupancy Prediction	Accuracy	The proportion of correct predictions (e.g., correctly classified comfort state or occupancy presence) among the total number of cases examined.	[133]
	F1-Score	The harmonic mean of precision and recall, providing a single score that balances both. Useful for imbalanced datasets (e.g., few “uncomfortable” instances).	
	Precision and Recall	Precision: of all the positive predictions, how many were actually correct? Recall: of all the actual positive cases, how many were correctly identified?	
Fault Detection and Diagnosis (FDD)	Accuracy	The overall correctness of the fault detection model (both faults and normal operations).	[134]
	False Alarm Rate (FAR)	The rate at which the system incorrectly identifies a fault when none exists. A critical metric for building operator trust.	
	Detection Rate (or Recall)	The proportion of actual faults that were correctly identified by the system.	
Reinforcement Learning (RL) Control	Cumulative Reward	The total reward accumulated by the agent over an episode. A higher reward generally indicates better performance against the defined objectives.	[135,136]
	Energy Savings (%)	The percentage reduction in energy consumption compared with a baseline controller (e.g., rule-based or thermostat).	
	Comfort Violations/PPD (%)	The amount of time or percentage of occupants predicted to be outside the comfort zone (as defined by ASHRAE 55).	

**Table 8:** Comparative analysis of advanced AI methods for smart building control.

Criteria	Classical RL	Deep RL	Multi-Objective RL	Digital Twin + RL
Energy Savings	10–20% [109,121]	15–25% [112,113]	20–30% cost reduction [121,126]	15–25% [112]
Implementation Complexity	Low [107]	High [112]	Very High [107]	Very High [112]
State Space Handling	5–15 variables [107,109]	20–100+ variables [35]	30–200+ variables [35,121]	100–1000+ variables [35]
Convergence Speed	100+ months [107]	5–24 K episodes [106,112]	2–10x slower than DRL [126]	Weeks–months with pre-training [112]
Real-time Deployment	Easy [107,109]	Complex [35,112]	Difficult [121,126]	Medium (after setup) [112]
Interpretability	High (Q-tables) [107]	Low (black box) [35]	Medium (Pareto solutions) [121,126]	High (physics-based) [112]

## Data Availability

Not applicable.

## Abbreviations

The following abbreviations are used in this manuscript:

AI	Artificial Intelligence
ML	Machine Learning
IEQ	Indoor Environmental Quality
HVAC	Heating, Ventilation, and Air Conditioning
IoT	Internet of Things
DRL	Deep Reinforcement Learning
DT	Digital Twin
DT-B	Digital Twin of the Building
DT-H	Digital Twin of the Human
LLM	Large Language Model
MORL	Multi-Objective Reinforcement Learning
RL	Reinforcement Learning
BIM	Building Information Modeling
PIR	Passive Infrared
mmWave	Millimeter Wave
CSI	Channel State Information
HR	Heart Rate
HRV	Heart Rate Variability
PMV	Predicted Mean Vote
GAN	Generative Adversarial Network
VAE	Variational Autoencoder
CV	Computer Vision
RNN	Recurrent Neural Network
CNN	Convolutional Neural Network
LSTM	Long Short-Term Memory
SVM	Support Vector Machine
SVR	Support Vector Regression
ANN	Artificial Neural Network
DQN	Deep Q-Network
DDPG	Deep Deterministic Policy Gradient
PPO	Proximal Policy Optimization
SAC	Soft Actor-Critic
XAI	Explainable Artificial Intelligence
XGBoost	eXtreme Gradient Boosting
SHAP	SHapley Additive exPlanations
LIME	Local Interpretable Model-Agnostic Explanations
ETL	Extract, Transform, Load
AutoML	Automated Machine Learning
NDN	Named Data Networking
WSN	Wireless Sensor Network
IIoT	Industrial Internet of Things
LPWAN	Low-Power Wide-Area Network
TEG	Thermoplastic Generator
SDN	Software-Defined Networking
SAX	Symbolic Aggregate approXimation
PCA	Principal Component Analysis
OPTICS	Ordering Points to Identify the Clustering Structure
API	Application Programming Interface
BAS	Building Automation System
TRIPOD-LLM	Transparent Reporting of a Multivariable Prediction Model for Individual Prognosis or Diagnosis—Large Language Model
HPC	High-Performance Computing
GPU	Graphics Processing Unit
NLP	Natural Language Processing
DNN	Deep Neural Network
SaaS	Software as a Service
SDK	Software Development Kit
TEG	Thermoelectric Generator
